# Molecular insights into the determinants of substrate specificity and efflux inhibition of the RND efflux pumps AcrB and AdeB

**DOI:** 10.1099/mic.0.001438

**Published:** 2024-02-15

**Authors:** Julia Wilhelm, Klaas Martinus Pos

**Affiliations:** ^1^​ Institute of Biochemistry, Goethe-University Frankfurt, Max-von-Laue-Str. 9, D-60438 Frankfurt am Main, Germany

**Keywords:** antibiotic resistance, efflux, efflux pump inhibitors, polyspecificity, Resistance Nodulation cell Division (RND), tripartite systems

## Abstract

Gram-negative bacterial members of the Resistance Nodulation and cell Division (RND) superfamily form tripartite efflux pump systems that span the cell envelope. One of the intriguing features of the multiple drug efflux members of this superfamily is their ability to recognize different classes of antibiotics, dyes, solvents, bile salts, and detergents. This review provides an overview of the molecular mechanisms of multiple drug efflux catalysed by the tripartite RND efflux system AcrAB-TolC from *Eschericha coli*. The determinants for sequential or simultaneous multiple substrate binding and efflux pump inhibitor binding are discussed. A comparison is made with the determinants for substrate binding of AdeB from *Acinetobacter baumannii*, which acts within the AdeABC multidrug efflux system. There is an apparent general similarity between the structures of AcrB and AdeB and their substrate specificity. However, the presence of distinct conformational states and different drug efflux capacities as revealed by single-particle cryo-EM and mutational analysis suggest that the drug binding and transport features exhibited by AcrB may not be directly extrapolated to the homolog AdeB efflux pump.

## Scope of this review

Multidrug resistance (MDR) is a major threat to human health. The WHO declared Gram-negative MDR *Acinetobacter baumannii*, *Pseudomonas aeruginosa*, and *Enterobacteriaceae* species top priority for research and development of new antibiotics [1, 2]. In the year 2019, infections caused by antibiotic-resistant bacteria were, after ischaemic heart disease and stroke, the most common cause of death in people of all ages with 1.3 million cases worldwide [3]. To understand and combat MDR, there is an urgent need to grasp the molecular basis of antimicrobial resistance for the development of new drugs and inhibitors of drug resistance mechanisms.

MDR is usually caused by a combination of different types of resistance mechanisms (see Darby *et al*. [4] for a recent review). This review will discuss multidrug resistance efflux pumps. These pumps belong to different superfamilies and families of transporters, which are briefly introduced. The focus will be on the Gram-negative bacterial members of the Resistance Nodulation and cell Division (RND) superfamily, in particular on those belonging to the hydrophobic/amphiphile exporter (HAE)−1 family. One of the intriguing features of the members of this pump family is their ability to recognize multiple drugs from different antibiotic classes, but also dyes, solvents, bile salts and detergents. Despite the overwhelming amount of data on known substrates for these pumps, many compounds that may also be substrates of these pumps are likely still unknown, since members of these HAE-1 transporters have also been recognized to be involved in processes such as quorum sensing, biofilm formation and virulence [5, 6]. The review will go in somewhat detail on the substrate polyspecificity of two RND pumps, AcrB from *Escherichia coli* and AdeB from *A. baumannii*, acting within the AcrAB-TolC or AdeABC multidrug efflux systems, respectively. In addition, recent examples of pump inhibition by efflux pump inhibitors (EPIs) will be addressed. Already comparing only these two well-studied efflux pumps will give insights in the flexibility of drug recognition and transport, and the conclusion that studying only one or two isolated efflux systems in a single bacterial species will not be adequate to answer the diverse properties of efflux pumps seen *in vivo*.

## Drug efflux pumps in the Gram-negative envelope setting

### Reduction of drug penetration and accumulation as intrinsic resistance mechanism

The cell envelope in Gram-negative bacteria comprises an inner membrane (IM), a periplasmic peptidoglycan cell wall, and an outer membrane (OM). Unlike the IM that is a symmetric phospholipid bilayer, the OM is composed of lipopolysaccharides (LPS) and phospholipids, in the outer and inner leaflet, respectively. The asymmetric nature of the OM constitutes an intrinsic permeability barrier for both hydrophobic and hydrophilic molecules, including drugs that need to reach their intracellular targets [7]. Large, and lipophilic molecules, such as macrolides antibiotics, rifamycins, novobiocin (NOV) and fusidic acid (FUA), enter the cells through (slow) penetration of the asymmetric OM bilayer and consequently show only poor activity against Gram-negative bacteria [8]. In contrast, smaller and more hydrophilic compounds, such as β-lactam antibiotics, readily enter the cell via the porin channels [8, 9], as those allow for the uptake of small, hydrophilic molecules (molecular mass cut-off ~600 Da) [10]. Hence, it is not surprising that reduced drug penetration due to alterations in porin expression was shown to be a major factor in β-lactam resistance in clinical isolates [10, 11]. These alterations include genetic mutation within the porin constriction zone (that defines channel size and ion selectivity), sequential replacement of porins, down-regulation of porin expression or even the complete loss of certain porins [10, 11]. Low membrane permeability in *P. aeruginosa* and *A. baumannii* is attributed to the expression of porins of unusually low permeability (OprF and OmpA_Ab_, respectively), designated ‘slow porins’, and plays a major role in high-level intrinsic antimicrobial resistance of these organisms [12].

**Fig. 1.**
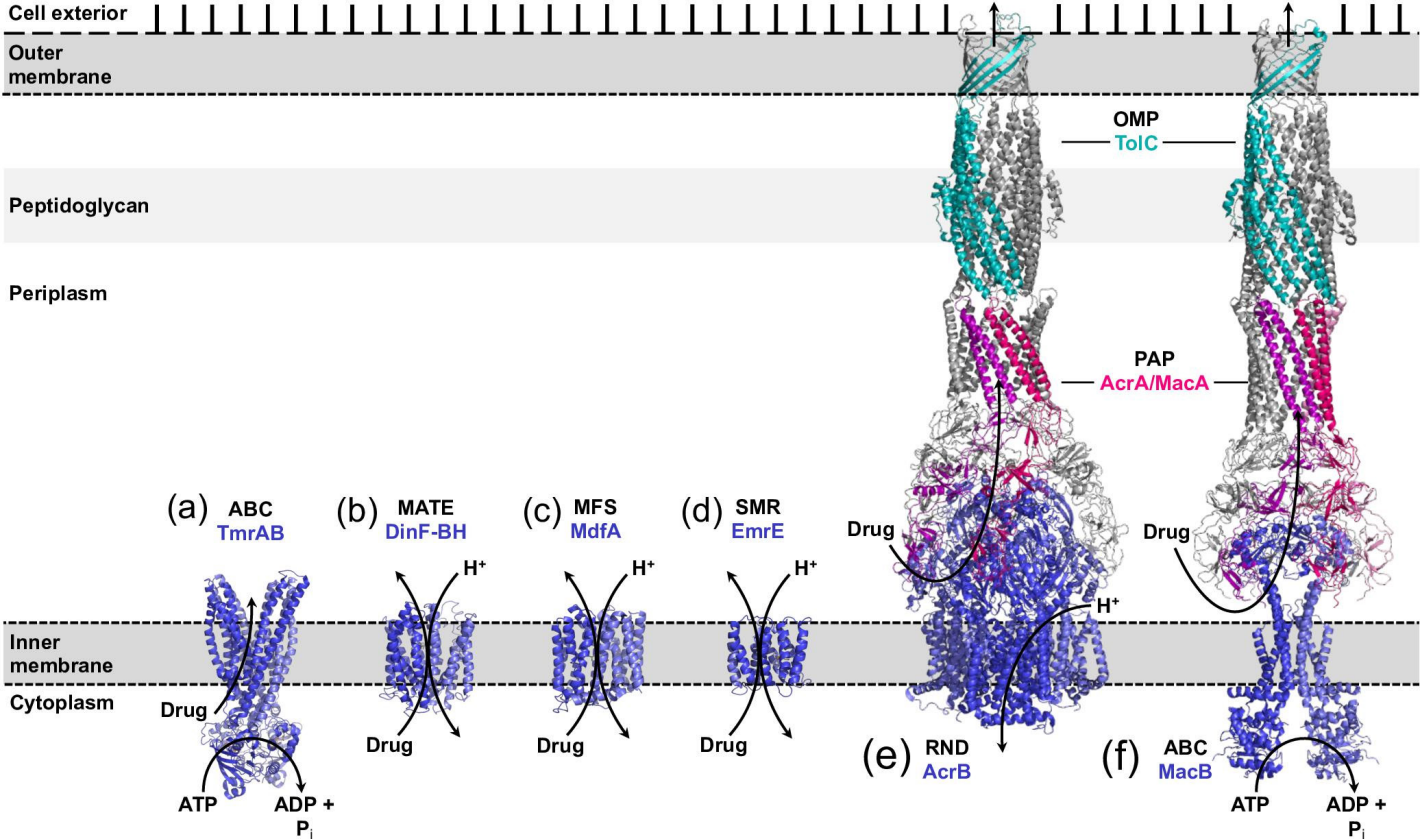
Schematic representation of common drug transporters within the cell envelope of Gram-negative bacteria. The cell envelope of Gram-negative bacteria has three layers: i) the asymmetric outer membrane, composed of lipopolysaccharides (LPS) and phospholipids in the outer and inner leaflet, respectively, ii) the peptidoglycan cell wall in the periplasm, and iii) the inner membrane. (**a–f**) Structures of representative inner membrane drug transporters are shown for five out of the six multidrug efflux transporter (super)families: ATP-binding cassette (ABC), represented by (**a**) TmrAB, (PDB ID: 6RAH) and (**f**) MacB, (PDB ID: 5NIK), (**b**) multidrug and toxic compound extrusion (MATE) (DinF-BH, PDB ID: 4LZ9), (**c**) major facilitator superfamily (MFS) (MdfA, PDB ID: 4ZOW), (**d**) small multidrug resistance (SMR) (EmrE, PDB ID: 3B5D), and (**e**) resistance, nodulation and cell division (RND) (AcrB, PDBID: 5O66). RND-type transporters (**e**) and occasionally also ABC-transporters (**f**) and MFS transporters (not shown) form tripartite complexes together with a periplasmic adaptor protein (PAP) and an outer membrane protein (OMP). Substrate transport via ABC-type pumps is driven by the binding and hydrolysis of ATP (primary transport), while the members of the other super(families) are secondary transporters that utilize the electrochemical gradient of ions across the inner membrane (H^+^ or Na^+^) to energize substrate transport. The structures of the inner membrane transporters, the PAPs and OMFPs are shown as cartoons in blue, pink/grey and teal/grey colours, respectively.

Multidrug efflux pumps that counteract the influx of antibiotics by active efflux, contribute further to bacterial intrinsic resistance (Fig. 1) [8, 13]. Constitutive expression of efflux pumps was reported to be the first line defence mechanism of bacterial cells facing antibiotic stress [14, 15]. In this way, the intracellular drug concentration is kept below its minimal inhibitory concentration (MIC), allowing for the acquisition of other long-term resistance determinants, such as changes in the expression of resistance-mediating genes and/or mutations [15]. In Gram-negative bacteria, the IM is spiked with transporters of various classes that function in a coordinated network to accomplish drug efflux in two consecutive steps [16] (Fig. 1). Translocation across the IM is performed by single-component transporters that recognize a rather limited but overlapping set of substances [16–18] (Fig. 1a–d). Subsequently, drug molecules are recognized and extruded by IM- and OM-spanning tripartite efflux systems comprising an OM factor/protein (OMF/OMP) and periplasmic membrane fusion protein (MFP, also designated as periplasmic adaptor protein, PAP) together with an IM transporter of the RND superfamily, or alternatively from the ABC-superfamily or MFS (see below) (Fig. 1e–f).

Despite examples for some toxins like tetracycline (TET) or ethidium (ETH) that show additive or synergistic effects [16, 19], other reports suggest that RND efflux pumps such as AcrAB-TolC in an *E. coli* strain devoid of any other efflux pump gene solely provide wild-type resistances against many toxins [17, 18, 20]. Upregulation of RND efflux pump gene expression, caused by mutations in the respective local repressor or global transcription factor regulators genes [21–25], is a major contributor to the MDR phenotype of clinical isolates of pathogenic bacteria (examples listed in Du *et al*. [26]). Moreover, mutations within the transporter genes may alter the RND substrate specificity and confer clinically relevant levels of MDR, as observed for a G288D substitution within the AcrB substrate binding pocket of *Salmonella typhimurium* [27].

### Tripartite complex assembly

**Fig. 2.**
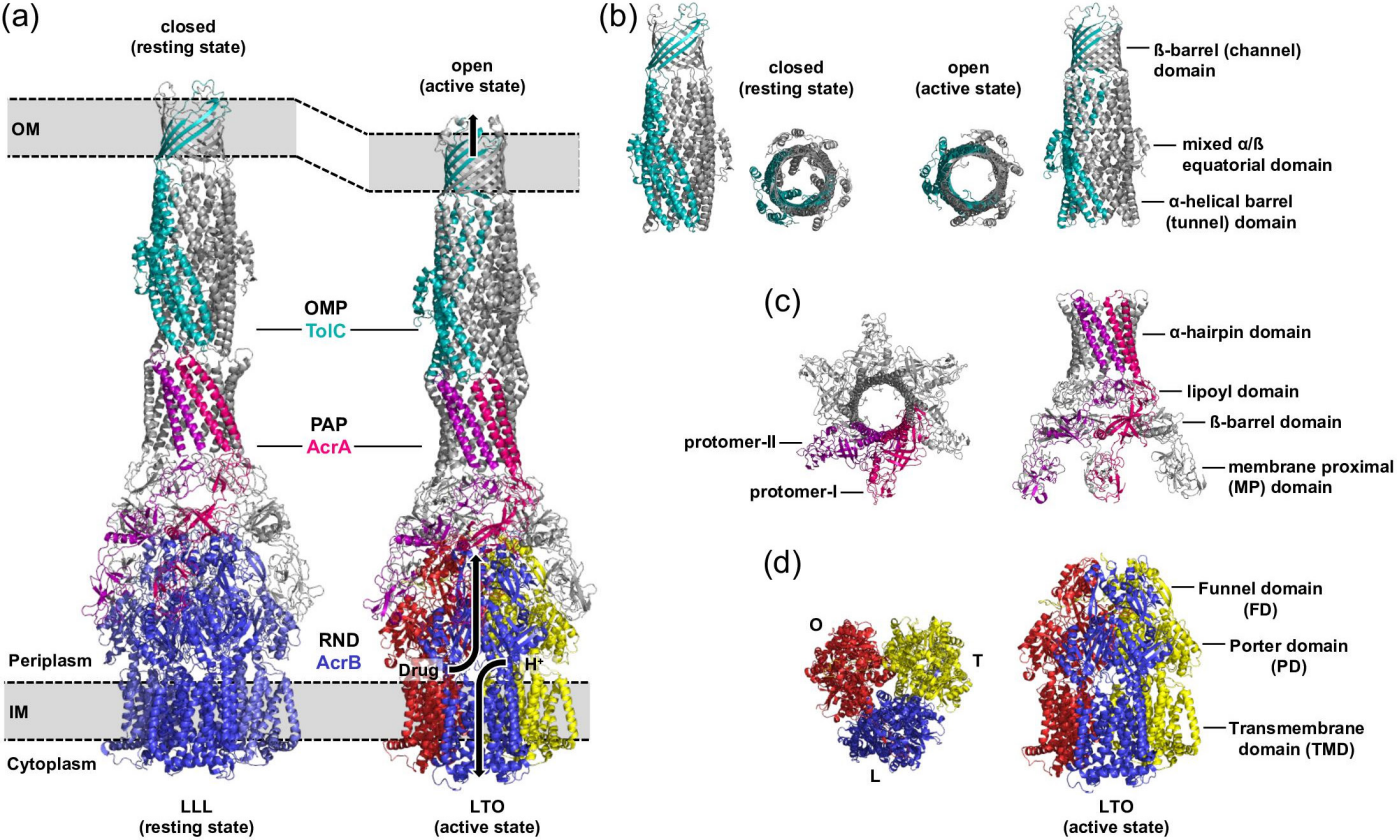
Tripartite complex assembly of the *E. coli* AcrAB-TolC multidrug efflux pump. (**a**) The trimeric outer membrane protein (OMP) TolC and the trimeric inner membrane RND transporter AcrB are connected via the hexameric periplasmic adaptor protein (PAP) AcrA, forming the tripartite complex AcrAB-TolC, that spans the entire cell envelope. Left: In the absence of substrates, AcrB presumably adopts the all-loose (LLL) conformation, representing the resting state of the pump. In the resting state, the AcrA hexamer is loosely packed and does not tightly seal the assembly towards the periplasm. The TolC β-barrel domain inside the outer membrane (OM) is open to the cell exterior, whereas the TolC α-helical barrel in the periplasm is closed (PDB ID: 5V5S). Right: In the presence of drug, AcrB predominantly adopts the asymmetric LTO conformation, i.e. an active state. In this state, the TolC α-barrel adopts an open conformation, enabling substrates to exit toward the cell exterior (PDB ID: 5O66). The direction of drug/proton antiport is indicated by black arrows. The change from the resting state to the active state appears to result in a compression of the tripartite pump system of approx. 10 Å. (**b**) Side and top views on the TolC trimer in the closed (left image) or open (right image) state. The TolC structure can be divided into a β-barrel and α-barrel domain, the latter including a mixed α/β-equatorial domain. For clarity, one protomer within the trimeric TolC assembly is highlighted in cyan. Opening/closing at the periplasmic end of TolC is achieved by an iris-like movement of the TolC coiled-coils, induced by repacking of AcrA. (**c**) Top and side views on the AcrA hexamer. For clarity, protomer-I and -II within the hexameric assembly are highlighted in purple and hotpink, respectively. The AcrA monomer can be structurally divided into four domains, i.e. the α-hairpin, lipoyl, β-barrel, and membrane proximal (MP) domains. (**d**) Top and side views on the asymmetric AcrB trimer. The structures of the loose (**l**), tight (**t**) and open (**o**) conformers are represented in blue, yellow, and red colour, respectively. An AcrB monomer can be structurally divided into three domains, the periplasmic funnel domain (FD), the porter domain (PD) and a 12 α-helices comprising transmembrane domain (TMD). This figure was adapted and modified fromFig. 2 in Kobylka, Kuth, Müller *et al.* [60].

Whereas the AcrB (Acriflavine resistance protein B) component in the AcrAB-TolC complex was recognized to be responsible for substrate recognition/transport and energy transduction [28–34], only the presence of the completely assembled tripartite multidrug efflux system confers a drug resistance phenotype. Recent studies indicated that also AcrA is involved in substrate binding and recognition [35]. *In vitro* tripartite AcrAB-TolC (Fig. 2) and homolog MexAB-OprM structures have been elucidated via single-particle transmission- or cryo-EM [36–39]. AcrAB-TolC has also shown to be complexed with a small membrane helix AcrZ, which interacts with the TMD of AcrB [40] and was demonstrated to modulate AcrAB-TolC activity for a subset of antibiotics [41]. It appears that the tight interactions between AcrB, AcrA and TolC not only provides a drug efflux conduit through the periplasm, but that conformational changes in AcrB are transduced to the asymmetric AcrA subunits (Fig. 2) which might lead to concertation of conformational change of the entire efflux pump system resulting in a directed catalysis of drug efflux [42]. It is, by the way, good to recall that in Gram-negative bacteria, efflux pumps act in synergy with the OM barrier and hence are particularly efficient in organisms that display low OM permeability [8, 13, 43, 44].

A recent review [42] highlights the intrinsic complexity and flexibility between the tripartite subunits and will not be further discussed here.

## Multidrug efflux pumps

### Multidrug efflux pump superfamilies

According to the transporter classification database (TCDB, www.tcdb.org) [45–47], transmembrane transporters involved in active efflux of antimicrobial agents are currently grouped into six distinct families – the ATP-binding cassette (ABC) superfamily, the multidrug and toxic compound extrusion (MATE) family, the major facilitator superfamily (MFS), the small multidrug resistance (SMR) family, the proteobacterial antimicrobial compound efflux (PACE) family, and the resistance, nodulation and cell division (RND) superfamily (Fig. 1). Whereas substrate transport via ABC-type pumps is driven by the binding and hydrolysis of ATP (primary active transport), the other five classes are secondary active transporters that utilize the electrochemical gradient across the IM to energize substrate transport [26]. The readers are referred to other reviews [26, 48–51] on members of the (super)family members mentioned above. Below, we will address the RND-type multidrug efflux pumps, with emphasis on AcrB and AdeB from *E. coli* and *A. baumannii*, respectively.

### Resistance, Nodulation and cell Division (RND) superfamily

Members of the Resistance, Nodulation, and cell Division (RND) (TCDB # 2.A.6) superfamily [52] are ubiquitously found in all domains of life and are involved in a variety of cellular processes [53]. Currently, the RND superfamily is grouped into nine phylogenetically distinct families [45, 46]: the heavy metal efflux (HME) family (TCDB # 2.A.6.1), the (largely Gram-negative bacterial) hydrophobe/amphiphile efflux-1 (HAE-1) family (TCDB # 2.A.6.2), the putative nodulation factor exporter (NFE) family (TCDB # 2.A.6.3), the SecDF (TCDB # 2.A.6.4) family whose members are involved in protein secretion [54] via the SecYEG translocon [53, 55], the (largely Gram-positive bacterial) hydrophobe/amphiphile efflux-2 (HAE-2) family (TCDB # 2.A.6.5), the eukaryotic sterol transporter (EST) family (TCDB # 2.A.6.6), the (largely archaeal) hydrophobe/amphiphile efflux-3 (HAE-3) family (TCDB # 2.A.6.7), the small, brominated, aryl polyene pigment exporter (APPE) family (TCDB # 2.A.6.8) and the dispatched family (TCDB # 2.A.6.9).

In Gram-negative bacteria, members of the HME (e.g. *E. coli* CusA) and HAE-1 (e.g. *E. coli* AcrB) families transport heavy metal ions and various antimicrobial drugs, respectively, and hence contribute to their resistance phenotype. Members of the HAE-2 (e.g. the *Mycobacterium tuberculosis* mycolic acid transporter MmpL3 [56, 57]) and HAE-3 families (e.g. the *Burkholderia multivorans* hopanoid transporter HpnN [58]) on the other hand, are involved in cell envelope biosynthesis and hence indirectly contribute to antimicrobial resistance [59].

**Fig. 3.**
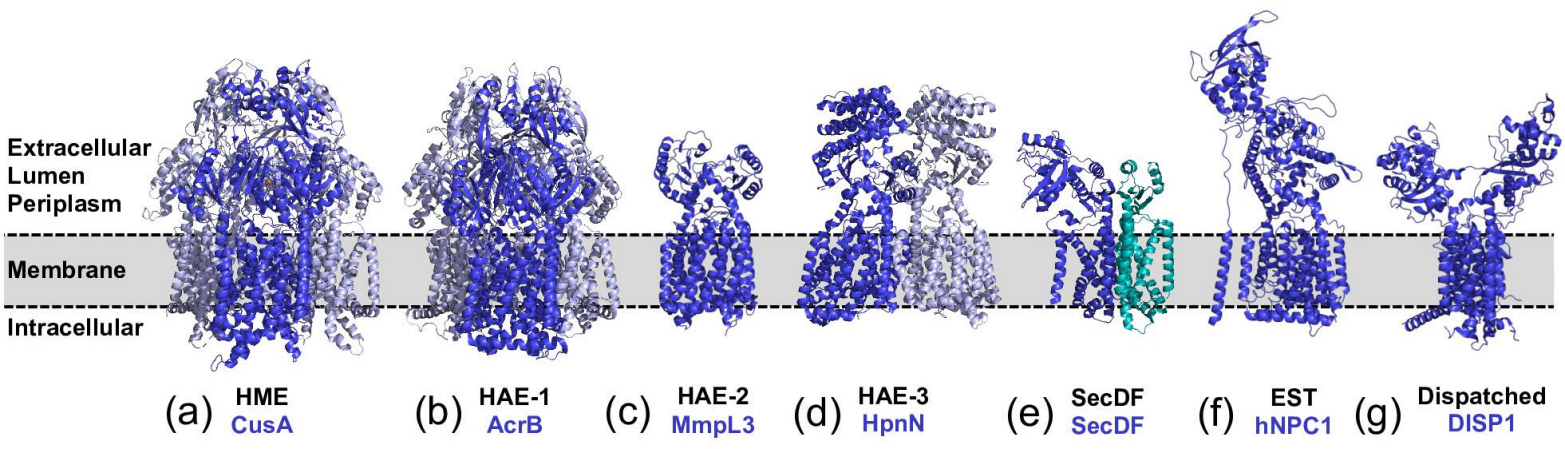
Overview of the structural diversity of transporter proteins within the RND superfamily. (**a–g**) Structures of representative transporters for seven out of the nine phylogenetic RND families are shown: (**a**) Structure of the homotrimeric Cu(I) and Ag(I)/H^+^ antiporter CusA from *Escherichia coli*, a member of the HME family (PDB ID: 3KSS). (**b**) Structure of the homotrimeric multidrug/H^+^ antiporter AcrB from *E. coli*, a HAE-1 family member (PDB ID: 4DX5). (**c**) Structure of monomeric mycolic acid transporter MmpL3 from *Mycobacterium smegmatis*, a member of the HAE-2 family (PDB ID: 6AJF). The structure lacks the encoded cytosolic domain that is suggested to mediate subcellular localization. (**d**) Structure of the dimeric hopanoid transporter HpnN from *Burkholderia multivorans*, a member of the HAE-3 family (PDB ID: 5KHN). (**e**) Structure of the heterodimeric *Thermus thermophilus* SecDF that is involved in protein secretion via the SecYEG translocon (PDB ID: 3AQ). (**f**) Structure of the monomeric human NPC1, a member of the eukaryotic sterol transporter (EST) family that, in conjunction with a small soluble protein NPC2, functions in cholesterol homeostasis (PDB ID: 3JD8). The human NPC1 features 13 TM helices of which TMH2-TMH13 exhibit the typical RND-fold. (**g**) Structure of the monomeric murine Dispatched homologue 1 (DISP1), a representative of the dispatched family, that plays an essential role in the hedgehog signalling pathway during embryogenesis and tissue regeneration (PDB ID: 7RPH).

Almost all bacterial RND proteins are proton motive force (pmf)-driven antiporters [42, 53, 60, 61], that feature a 12 transmembrane helices (TMH) comprising transmembrane domain (TMD) and a soluble portion formed by two extracellular loops of variable size. The TMD harbours residues essential for proton translocation [32–34, 62–64] and its structural fold is quite conserved among the different families [53]. The soluble portion on the other hand, harbours the substrate binding site(s) and, not surprisingly, greatly varies between the families [53, 61]. The structural and functional diversity within the RND superfamily, including some of eukaryotic origin, is illustrated in Fig. 3.

### HAE-1 efflux pump systems

The members of the HAE-1 family from Gram-negative bacteria work in conjunction with a periplasmic membrane fusion protein (MFP, also defined as periplasmic adaptor protein, PAP) and an OM factor/protein (OMF/OMP). RND, PAP, and OMF/OMP assemble in tripartite complexes, forming elongated, in part channel-containing structures that span the entire cell envelope [36, 37, 40, 42, 65–68] (Figs 1e and 2). The RND component constitutes the core component of the tripartite complex, as it is determinant for both substrate specificity [28–30] and energy transduction [32, 33, 62, 63, 69]. The presence of all three complex components is required for drug efflux activity *in vivo* [13]. HAE1-type transporters recognize and extrude an extraordinary wide range of structurally distinct compounds, which they capture from the periplasm and the outer leaflet of the IM [13]. Drug efflux is largely energized by the proton-motive force [60], although VexF from *Vibrio cholerae* was reported to be sodium-ion-dependent [70]. HAE-1-type proteins mostly function as homotrimers. There are notable exceptions such as the *E. coli* MdtBC, which was shown to assemble as heterodimer (B_2_C) [71]. In the latter heterotrimer, only the C subunit appears to bind and export substrates, whereas the B subunits appear to induce the structural changes needed for substrate transport upon proton translocation [72].

The first crystallographic structure of a HAE1-type multidrug transporter was the *E. coli* AcrB [62] (see below). Further structural information on two other HAE1-type proteins became available in the following decennium, namely *P. aeruginosa* MexB [73] and *Neisseria gonorrhoeae* MtrD [74]. Later, structures of *Campylobacter jejuni* CmeB [75], *A. baumannii* AdeB [76–78]*, S. thyphimurium* AcrB [79], *P. aeruginosa* TriC [80], *N. gonorrhoeae* MtrD (substrate-bound) [81], *A. baumannii* AdeJ [82], and very recently AcrD from *E. coli* [83], OqxB from *Klebsiella pneumoniae* [84], BpeF and BpeB from *Burkholderia pseudomalleii* [85] were reported. These proteins were shown to adopt the same overall fold as *E. coli* AcrB, which (to date) is one of the best studied members of the HAE-1 family.

### HAE-1 efflux pump systems in *Escherichia coli*

The ‘efflux network’ in *E. coli* was initially investigated by individual chromosomal knock-out of 16 efflux pumps genes [17]. Another study tested the activity of 37 individual efflux pumps by overexpression of their genes in *E. coli*Δ*acrAB* [18]. The outcome of both studies was that AcrAB-TolC acts as the major efflux pump in *E. coli* conferring resistance to multiple drugs. *E. coli* Δ*tolC* is often used as efflux-deficient mutant, since it causes the inactivation of all RND drug efflux systems in *E. coli* (AcrAB-TolC, AcrAD-TolC, MdtABC-TolC, MdtEF-TolC) and of ABC-transporter-type complexes MacAB-TolC, YhbFGSR-TolC, and HlyBD-TolC as well as the MFS-type complexes EmrAB-TolC and EmrKY-TolC [42]. However, *tolC* inactivation leads to pleiotropic effects not necessarily linked to the loss of drug efflux pump function [20, 86]. Recently, Teelucksingh *et al.* [20] reported an efflux platform based on a designed *E. coli* strain devoid of 35 drug efflux genes (EKO-35). EKO-35 (which harbours the *tolC* gene) showed a substantially different phenotype compared to the Δ*tolC* strain under nutrient limitation. The phenotypical deviations observed between the two strains included a different pattern of drug sensitivity for the Δ*tolC* strain and increased susceptibility toward extreme acidic or alkaline conditions. EKO-35 showed in addition an increased biofilm formation compared to wild-type *E. coli*, which was enhanced by the co-expression RspA, a starvation-sensing protein. The EKO-35 strain was used for complementation by chromosomally encoded RND efflux systems and characterized against multiple drugs, which led to an overall substrate profiling of efflux pump polyspecificity. These studies again showed the supremacy of AcrAB-TolC with respect to the overall polyspecificity. Other tripartite HAE-1 transporters, such as AcrEF-TolC and MdtEF-TolC appeared rather to be backup pumps, with overlapping substrate specificities compared to AcrAB-TolC. MdtEF-TolC is a special case, as it is overproduced under anaerobic conditions [87] and is most likely involved in the extrusion of nitrosyl-indol derivatives which are produced during nitrate respiration [88]. The AcrAD-TolC efflux pump was previously defined as aminoglycoside pump [89], but in the efflux platform, aminoglycoside susceptibility was not altered in the presence of this pump [20]. The recent single-particle cryo-EM structure of AcrD in complex with gentamycin, on the other hand, suggests a unique binding site for an aminoglycoside to bind within the central cavity of the AcrD trimer [83]. An AcrD substitution analysis of side chains involved in the apparent gentamycin binding indicated effects of the single-site-substitution variants on the gentamycin susceptibility. On bases of the analysis of the size and hydrophilicity of its drug binding pockets, AcrAD-TolC was predicted to be a pump for more hydrophilic substrates compared to its AcrAB-TolC counterpart [90]. Clearly, AcrD appears to have a higher propensity to efflux more hydrophilic, dianionic β-lactams [91] compared to AcrAB-TolC [92], but it also transports the very hydrophobic compounds FUA [20, 91], NOV, SDS, and bile salts [20].

The OM permeability is another factor which should be taken into account as it has considerable effect on the substrate specificity profile of a pump and the extent of resistance. This important aspect has been intensively addressed by the lab of Helen Zgurskaya [43, 44, 93] and has been considered in the efflux platform where ‘porinated’ EKO-35 strains were used as control.

To summarize, one of the many interesting findings made using the efflux platform, was the complementation of EKO-35 with only the AcrAB-TolC efflux system resulting in the restoration of the *E. coli* wild-type drug resistance phenotype [20]. This observation has implications on the hypothesis that Gram-negative cells such as *E. coli* may use a network of efflux pumps for drug resistance, consisting of single component drug transporters from the MFS or SMR-family to supply the tripartite RND-systems with drugs inside the periplasm (or outer leaflet of the IM). Nevertheless, for some toxins like ETH or antibiotics like TET, the combination of the activity of a single-component drug efflux pumps (MFS or SMR-type) and a tripartite RND efflux pump has led to an additional resistance effect [16, 19, 94, 95]. Whether this functional interaction is synergistic rather than additive in nature is debated and needs further experimentation.

## The *E. coli* multidrug efflux pump AcrB

### The architecture of the *E. coli* inner membrane RND transporter AcrB

**Fig. 4.**
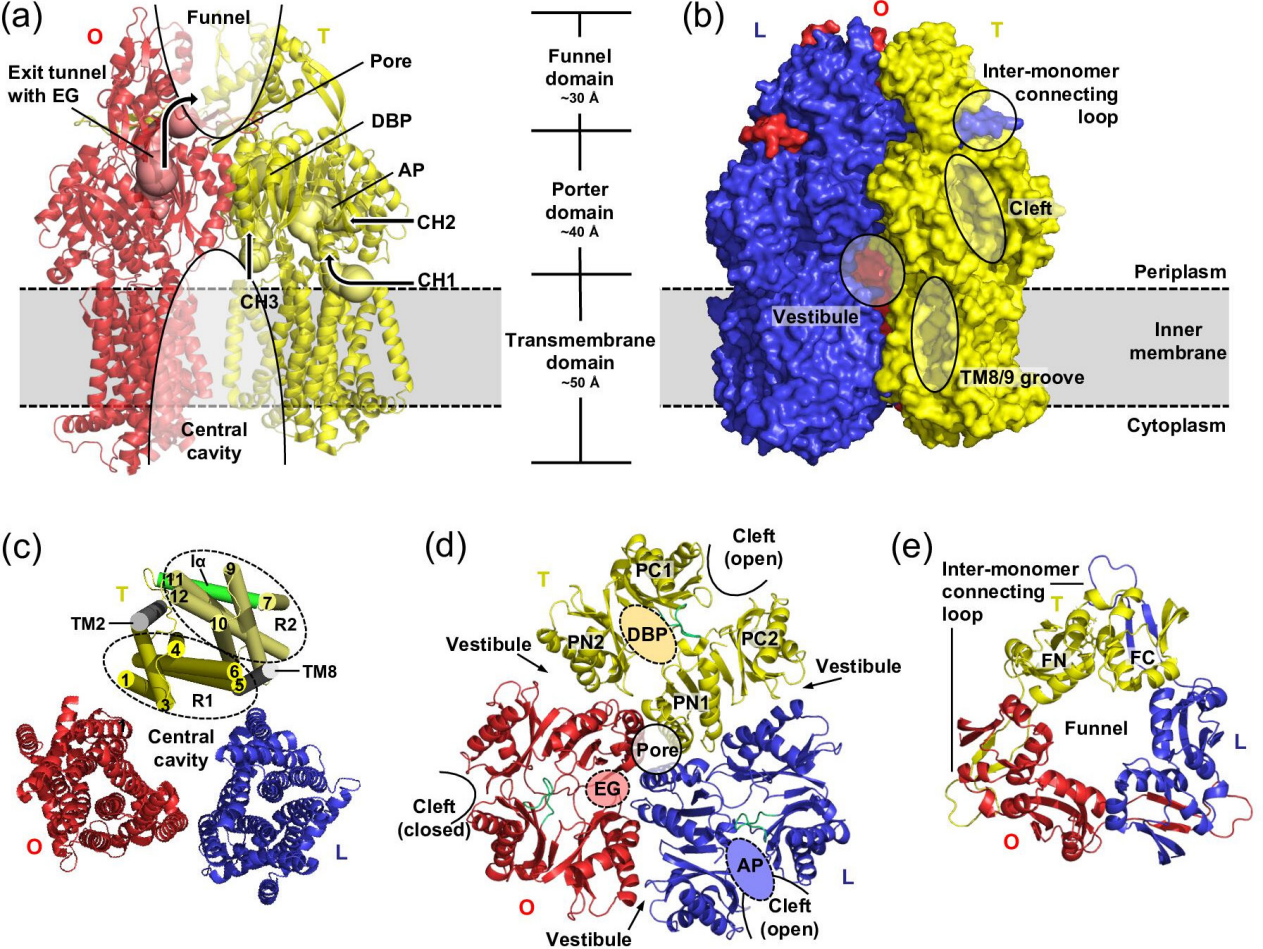
Architecture of the inner membrane RND transporter AcrB. (**a**) Side view on the AcrB T (yellow colour) and O (red) protomers is shown in cartoon representation. In this view, the L protomer is removed for a clearer view. The AcrB monomer can be structurally divided into three domains, the 12 α-helices comprising transmembrane domain (TMD) (50 Å), and the periplasmic porter (PD) (40 Å) and funnel domains (FD) (30 Å). The AcrB trimer comprises a distal funnel that narrows down to a central pore structure in the PD at about 15 Å above the membrane plane. Below this pore structure, there is a large central cavity, that further leads to a wide transmembrane hole, presumably filled with phospholipids. Within the L and T states, a tunnel system (CH1-CH4) grants access to the periplasmic substrate binding pockets, i.e. the access pocket (AP) and the deep binding pocket (DBP). Drugs can enter from the outer leaflet of the inner membrane via CH1 (entry at the TMH7/TMH8 groove) and via CH2 (entry from the periplasm). In the T state, CH3 and CH4 (which is not shown) provide direct access to the DBP via the central cavity and via the TMH1/TMH2 groove, respectively. The O protomer on the other hand, comprises no entry channels but features an exit tunnel with an exit gate (EG) that leads from the closed DBP towards the AcrB distal funnel. (**b**) Side view on the asymmetric (LTO) AcrB trimer in surface representation. The vestibule leading to the central cavity, the periplasmic cleft (entrance to CH2) and the TMH8/TMH9 groove (entrance to CH1) are highlighted. (**c**) Top view on the TMD. Within the TMD, each monomer consists of two structural repeats R1 (N-terminal region, cylindrical helices coloured in yellow, comprises TMH1 and TMH3-TMH6) and R2 (pale yellow, C-terminal region, comprises TMH7 and TMH9-TMH12). R1 and R2 are connected by a cytosolic α-helix (Iα, coloured in green) and are flanked by TMH2 and TMH8 (coloured in grey). The latter two helices link the TMD with the PD. TMH4 and TMH10 are situated in the centre of the R1 and R2 helical bundles, respectively, and harbour the side chains for the proton relay triad involved in proton translocation across the inner membrane, i.e. D407, D408 on TMH4 and K940 on TMH10. (**d**) Top view on the PD, that can be structurally subdivided into the N-terminal PN1 and PN2 subdomains and the C-terminal PC1 and PC2 subdomains. These subdomains form the two structural PN1/PC2 and PN2/PC1 units. In the L and T protomer, the PC1 and PC2 subdomains shape the AP. The PN2 and PC1 subdomains flank the DBP, which is exclusively present in an open state in the T protomer. The AP and DBP are separated by the switch loop, highlighted in green. In the O conformation, the periplasmic cleft and both binding pockets are closed, but the EG is open. (**e**) Top view on the FD. The N-terminal subdomain (FN) harbours a 35 Å-long hairpin structure, that is named inter-monomer connecting loop. It extends into the FC subdomain of the adjacent protomer. The different conformations of the asymmetric AcrB trimer are represented in blue (L, loose), yellow (T, tight) and red (O, open) (PDB ID: 4DX5). This figure was adapted and modified based on Fig. 3 from Kobylka, Kuth, Müller *et al.* [60].

**Fig. 5.**
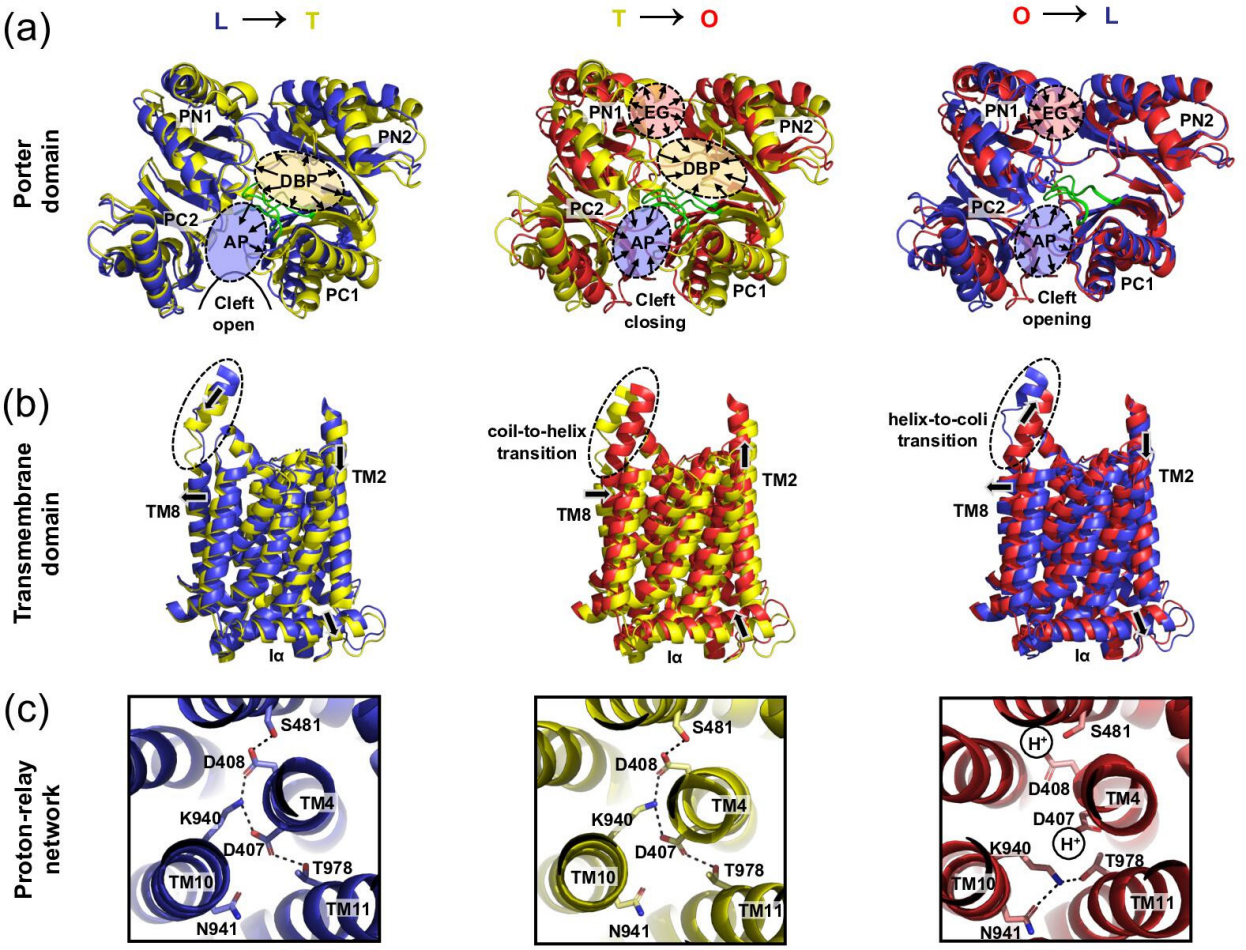
Structural basis of functional rotation and AcrB drug/proton antiport coupling mechanism. Drug/proton antiport is achieved by a concerted functional rotation of AcrB protomers through the three consecutive conformational states loose (L), tight (T) and open (O). Substrate binding occurs within the periplasmic porter domain (PD) and proton binding within the transmembrane domain (TMD). Structural rearrangements within the TMD and periplasmic PD are coupled via TMH2, connected to the PN1/PC2 subdomains unit and via TMH8, which is connected to the PN2/PC1 subdomains unit and includes the DBP. (**a**) Top view on the periplasmic PD. Structural changes within the PD during LTO transition are visualized by superimposition of the L and T, T/O and O/L conformations. (**b**) Side view on the TMD. Structural changes within the TMD during LTO transition are visualized by superimposition of L and T, T/O and O/L conformations. (**c**) Close up top view on the proton relay network within the TMD (TMH4, TMH10, and TMH11) in the L, T, and O conformations. In panels (a–c), the L conformer is shown in blue colour, T in yellow and O in red. In panel (a), the switch loop is highlighted in green. The model used for the superimpositions is PDB ID: 4D×5. Figures in panel (a) and (b) were adapted and modified from Fig. 4b in Kobylka, Kuth, Müller *et al.* [60].

AcrAB-TolC confers resistance to bacterial cells against multiple drugs and the property to recognize drugs as substrates and to energize drug efflux is contained in the IM component AcrB. The first structure of *E. coli* AcrB (113 kDa [1049 aa]) was solved at 3.5 Å resolution from crystals belonging to the trigonal space group R32 [62]. This structure depicts unliganded homotrimeric AcrB in a symmetric conformation, in which all protomers adopt the so-called loose (L) state (LLL-trimer conformation), presumably representing the resting state of the pump (Fig. 2) [34, 96]. The AcrB monomer can be structurally divided into a 12 α-helices comprising TMD and a large soluble portion (Fig. 4), formed by two periplasmic loops between TMH1/TMH2 and TMH7/TMH8, respectively. The large soluble portion can be further subdivided into the porter or formerly also called pore domain (PD) and the funnel domain (FD) [62]. Within the TMD, pseudo two-fold symmetry creates two structural repeats of TMH bundles, repeat 1 (R1, N-terminal, TMH1/TMH3-TMH6) and R2 (C-terminal, TMH7/TMH9-TMH12), that are connected by an extra-membrane α-helix (Iα) located parallel to the cytoplasmic membrane surface [62, 63] (Fig. 4c). TMH4 (R1) and TMH10 (R2), situated in the centre of the TMH bundles, harbour three titratable residues, D407, D408 (both on TMH4) and K940 (TMH10), that have been shown to be essential for AcrB activity and are part of the proton translocation network [32, 33, 62, 63, 69] (Fig. 5). R1 and R2 are each flanked by a single TM helix, TMH2 and TMH8, respectively. TMH1, TMH2, TMH7 and TMH8 connect the TMD and PD (Fig. 5a, b). The PD is divided into the N-terminal PN1 and PN2 and the C-terminal PC1 and PC2 subdomains, that also form two structural repeats. The PN1/PC2 repeat is linked to the TMD via TMH8 and PN2/PC1 repeat is connected to TMH2. Both structural repeats are composed of two similar α/β motifs connected by a shared β-strand [62, 63] (Fig. 4d). In the PN2 and PC2 subdomains, the motif is interrupted to form the FD-subdomains FN and FC, respectively [62] (Figs 4e and 5a). The FN subdomain of each AcrB protomer harbours a long hairpin structure (length 35 Å), the so-called inter-monomer connecting loop, that extends into the FC subdomain of an adjacent protomer and is a major factor for trimer stability [62, 97, 98]. The protomer arrangement within the AcrB trimer creates a 16 Å wide ring-like funnel starting at the distal end of AcrB and narrows down to a central pore-like structure in the PD about 15 Å above the membrane plane [62] (Fig. 4a, d, e). The diameter of this pore is defined by three closely interacting α-helices (Nα2 of the PN1 subdomain), which were initially designated as pore helices [62] (Fig. 4d). The pore was later to be found not a drug-conducting pore as the side chains of each of the pore helices always interact via H-bonding and no channel is formed which would allow for drug passage [99, 100]. Below the pore toward the membrane plane, the trimeric AcrB protomer arrangement creates a large central cavity (Fig. 4a, c). The TMD helical bundles of each protomer form below the central cavity a 30 Å wide TM hole that is filled with phospholipids [62, 101]. Between the central cavity and the TM hole, just above the membrane plane, so called vestibules are located at each of the three inter-monomeric PN2/PC2 subdomain interfaces. These vestibules form putative pathways for solutes to exchange between the periplasm and the central cavity [62] (Fig. 4b).

### Drug transport by AcrAB-TolC – the AcrB functional rotation mechanism

Following the initial symmetric structures of AcrB (LLL conformation), several groups reported asymmetric structures of AcrB, derived from crystals belonging to the monoclinic (C2, 2.8–2.9 Å (apo) [102, 103] and 3.1–3.3 Å (liganded) [102]), triclinic (P1, 3.0 Å) [103] and orthorhombic (P2_1_2_1_2_1_, 2.5 Å) [104] space groups. Within the asymmetric trimer, each of the three protomers adopts a different conformation designated as loose (L, or access), tight (T, or binding) and open (O, or extrusion) (LTO) [102, 103] (Fig. 4). Co-structures of substrates bound within the periplasmic part of AcrB [102, 105, 106] led to the identification of two voluminous periplasmic binding pockets, the access pocket (AP) [106] or also called the proximal pocket [105], and the deep or distal binding pocket (DBP) [102, 105]) (Fig. 4a, d). The AP and DBP are separated by an 11 amino acid comprising loop motif, designated as the switch loop [105, 106] (Fig. 4d). The different protomer conformations observed in the asymmetric (LTO) AcrB structures have been postulated to represent consecutive states of a transport cycle in which drug efflux through a tunnel system (see below) is accomplished via functional rotation [102, 103] in analogy to the binding change mechanism (or alternating site mechanism) of the F_1_F_o_ ATPase [107]. In brief, the suggested transport cycle starts with drug binding to a low affinity binding site (the AP) in the L state [105, 106] accessible via the periplasmic cleft formed by the PC1/PC2 subdomains. Upon L to T transition, conformational change of the PN2/PC1 subdomains open the DBP. Substrate is translocated to the DBP, supported by a conformational swing of the switch loop [106]. Subsequently, the energy-dependent T to O transition results in the collapse of the periplasmic binding pockets and a concomitant opening of an exit tunnel or exit gate (EG) (Figs 4a, d and 5a). In this way, substrates are squeezed out of the DBP and into the central funnel that guides the drug toward the AcrA and TolC tunnels (and finally to the cell exterior across the OM) [103]. To complete the catalytic cycle, conversion of O to L restarts the transport cycle [42, 63, 103]. The proposed mechanism implies a concerted conformational change of all protomers [99, 102–104]. Site-directed disulphide cross-linking experiments [69, 99] demonstrated that protomer subunit conformational flexibility is indispensable for AcrB activity. Moreover, inactivation of a single protomer within a fused AcrB trimer completely abolished transport activity [108]. If the protomers would cycle through the states L, T and O in an independent manner, i.e. regardless of the conformational states of the other protomers within the trimer, inactivation of one protomer would not lead to an inactive trimer. In analogy to the F_1_F_o_ ATPase binding change mechanism [107], it was furthermore postulated that the energy-dependent conversion of T to O might occur via bi-site activation [96]. Indeed, measurements of AcrB transport kinetics [109, 110] showed positive cooperativity in efflux catalysis for a set of β-lactam compounds. This might be due to bi-site activation via drug binding to neighbouring protomers, but cooperative effects within a single protomer, e.g. multiple drug binding, might be responsible for this observation as well. Further experiments also indicated that AcrB is able to adopt intermediate asymmetric states (e.g. LLT, LTT and TTO) besides the LTO state throughout the transport cycle [96, 111]. The site-directed disulphide cross-linking experiments, reported above, indicated that the AcrB trimer provides the conformational flexibility required to comprise more than one protomer in the same state within the trimer [99]. Moreover, single-particle cryo-EM analysis showed that AcrB can adopt intermediate conformational states within the AcrAB-TolC tripartite complex in the presence of the EPI MBX3132 [37]. The classification of the particles displayed different trimeric states, such as the TTT state (in 73 % of all particles), but also other states like LLL (1.4 %), LLT (6.2 %), and LTT (18.6 %). A structure of AcrAB-TolC determined in the same manner in the presence of drug substrate yielded exclusively LTO conformers [37]. The latter observation is consistent with the hypothesis of that the LTO trimeric state represents the lowest energy form in the presence of a drug substrate [96, 112]. In another study, the quantification of the steric clashes between protomers in hypothetical trimer models revealed that most AcrB trimer constellations that include more than one O states would be energetically unfavourable [63, 96, 111]. Nevertheless, whereas symmetric structures in which all protomers adopt the O conformation (OOO trimer) have not been observed for AcrB thus far, OOO states were recently reported for *A. baumannii* AdeB [76–78], *P. aeruginosa* TriC [80] and *C. jejuni* CmeB [75].

### AcrB tunnel system

Computational analysis of the asymmetric AcrB trimer structures revealed a tunnel system comprised of four channels [60, 103–105, 113–115], that grants access to the AP and/or DBP from the outer leaflet of the IM and/or the periplasm, and in addition an exit tunnel with the EG. Channel 1 (CH1) starts at the TMH7/TMH8 groove and merges with CH2 in the AP (Fig. 4a). CH2 permits access to the AP via a periplasmic cleft, confined by the PC1 and PC2 subdomains. In the T conformation this tunnel protrudes into the hydrophobic DBP formed by the PN2/PC1 subdomain interface at the distal site. CH3 connects the central cavity directly to the DBP, and CH4 starts at the TMH1/TMH2 groove and grants direct access to the DBP via the PC1/PN2 down pathway [112]. Both CH3 and CH4 bypass the AP and the switch loop. Within the T conformation, the channels are limited in length, up to the DBP. The DBP is a cul-de-sac due to distinct orientation of the PN1 and PN2 subdomains and the block created by the tilting of the PN1 subdomain of the adjacent O protomer. In the O conformation, the periplasmic access to all channels, the periplasmic cleft, and the substrate binding pockets are closed, while an exit tunnel from the closed DBP toward the central funnel of AcrB is now apparent (Fig. 4a). Of note, the channels CH1–CH4 are considered flexible entities during the LTO conformational cycle. By guiding drugs through AcrB from their entry regions toward the DBP in the L and T transition, and from the DBP through the EG toward the AcrB funnel in the T to O transition, the mechanism appears to mimic a peristaltic pump [103]. In line with this hypothesis, an intermediate AcrB/dodecylmaltoside (DDM) co-structure features trapped DDM, an AcrB substrate, in CH1 of the L protomer on its way toward the closed DBP. In this co-structure the entrance of CH1 is already closed due TMH8 tilting to prevent reverse transport toward the periplasm [116].

### Energy transduction and the alternating access drug/proton antiport mechanism

Drug efflux via AcrB is driven by proton motive force [13, 117]. In contrast to other secondary active transporters, RND-type proteins display a considerable spatial separation (approx. 50 Å) between proton relay network within the TMD that drives active efflux and substrate translocation sites within the PD. Three titratable residues within the TMD, i.e. D407, D408 on TMH4, and K940 on TMH10, in addition to R971 on TMH11, are part of the proton translocation network and were shown to be essential for AcrB activity (Figs 4c and 5c) [32, 33, 62, 63, 69].

During the LTO transitions the PN2/PC1 subdomains, comprising the DBP, undergo a significant conformational change relative to each other, especially during the L to T and T to O transitions (Fig. 5a) [63]. These changes constitute reorientations of the PN2 and PC1 subdomains while the individual subdomain α/β motifs move as rigid bodies [63, 102–104]. The structural rearrangements within the PD appear to be transduced to the TMD via TMH2 and TMH8, that function as coupling elements to the PN2/PC1 and PN1/PC2 subdomain units [63], respectively (Fig. 5b). Insights from high resolution X-ray crystallographic structures of AcrB wild-type and proton relay substitution variants D407N, D408N, K940A or R971A combined with molecular dynamics simulations led to the proposal of an alternating-access drug/proton antiport mechanism [63]. As described above, the transport cycle starts with drug binding to a low affinity binding site inside the AP [105, 106], which is accessible via the periplasmic cleft, constituted by the subdomains PC1 and PC2, that is open in the L and T state (Fig. 5a). At this stage, the three titratable residues D407, D408 in the R1 repeat and K940 in R2 within the TMD are ionized and engaged in salt bridges [62, 63] (Fig. 5c). Upon L to T transition, the substrate is translocated to the DBP, accommodated by structural reorientations within the PN2/PC1 subdomain unit and a conformational swing of the switch loop [106] (Fig. 5a). These changes cause a downshift of TMH2, a movement transduced to the Iα helix and the entire R1 repeat [63, 114] (Fig. 5b). The D407, D408 and K940 triad remains engaged and appears to act as hinge for the relative reorientation of R1 and R2 [63] (Fig. 5c). The R1 repeat movement leads to an alternating access pathway for protons from either side of the IM. Whereas the triad is exclusively accessible from the cytoplasmic side in the L conformation, in the T state, upon the TMH2 and R1 repeat shift, protons can enter from the periplasmic side, mediated by water channels as is visible in the X-ray structures and predicted by MD simulations [63]. The resulting protonation of D407 and/or D408 [33] causes a change in electrostatics between D407/D408 and K940. Since these residues are the located between the R1 and R2 structural repeats, R1 and R2 become disengaged. This disengagement results in a reorientation of K940 toward residues on TMH10 and TMH11 [63] and a lateral movement of R2, together with the Iα helix, resulting in an upshift of TMH2 [63]. In addition, a coil-to-helix transition and bending of the N-terminal part of TMH8, the so-called hoisting loop [118], is observed [102–104] (Fig. 5b). The coil-to-helix transition coincides with a movement of the PN1/PC2 subdomain unit relative to the PN2/PC1 subdomain unit and results in the closure of the periplasmic cleft and the AP (Fig. 5a). During the T to O transition, three concurrent subdomain movements are apparent: (i) The conformational flexibility of the PN2 and PC1 subdomains within the PN2/PC1 subdomain unit leads to a collapse of the periplasmic DBP, ii) The relative motions of the PC1 and PC2 subdomains close the AP and the periplasmic cleft, iii) The tilting of the PN1 subdomain toward the neighbouring T state protomer at the centre of the AcrB trimer results in the creation of the exit tunnel (Figs 4a and 5a). Due to these concerted movements, drug substrates are guided from the DBP into the AcrB central funnel and toward AcrA and TolC. Upon O to L transition, a proton or protons are released from D407 and/or D408 into the cytosol (Fig. 5c). This change in electrostatics in the TMD leads to re-engagement of R1 and R2 and a helix-to-coil transition of the N-terminal portion of TMH8 (Fig. 5b). TMH8 is connected to the PN1/PC2 subdomain unit, which concomitantly alters its conformation, resulting in the closure of the exit tunnel (and EG) and the opening of the periplasmic cleft and AP (Fig. 5a, c). The AcrB protomer is thus returned in its L conformation, initiating a restart of the transport cycle [60]. The number of protons transported per LTO cycle is not known thus far. Suggestions range between one [119, 120] or two protons [42, 63] per cycle. Another open question is the drug/proton stoichiometry, which is particularly difficult to address, since the drugs are most likely captured from the periplasm or the outer leaflet of the IM, and therefore, in contrast to the protons, not transported across the IM [13]. Since AcrB has multiple binding sites [105, 116], it is likewise also unclear, how many drug molecules can be captured and transported during one LTO cycle.

### Molecular basis for multidrug recognition and transport

**Fig. 6.**
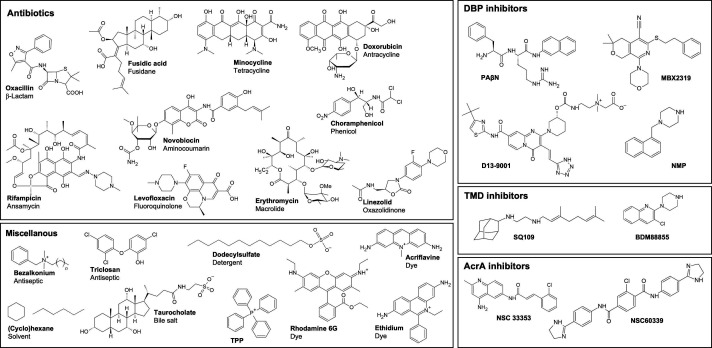
Substrates of the AcrAB-TolC efflux system and efflux pump inhibitors. The substrate spectrum of AcrB comprises a variety of structurally diverse compounds, including different classes of antibiotics (β-lactams, tetracyclines, fluoroquinolones macrolides, antracyclins, aminocoumarins, ansamycins, phenicols, oxazolidinones, but not aminoglycosides), dyes (such as acriflavine, rhodamine 6G, ethidium), antiseptics (such as benzalkonium, triclosan), simple solvents (e.g. hexane), detergents (e.g. dodecylsulfate) and bile salts (e.g. taurocholate). Efflux pump inhibitors binding to the deep binding pocket (DBP inhibitors) are phenylalanylarginine-β-naphthylamide (PAβN), the pyranopyridine MBX2319, the pyridopyrimidine D13-9001, and 1-(1-naphthylmethyl)-piperazine (NMP). Inhibitors binding to the transmembrane domain of RND efflux pumps are the 1,2-ethylenediamine SQ109 (targets MmpL3 from *Mycobacterium smegmatis* and *Mycobacterium tuberculosis*) and the pyridylpiperazin BDM88855 (targets AcrB from *E. coli* and *K. pneumoniae*). Inhibitors of the periplasmic adaptor protein component AcrA of the tripartite AcrAB-TolC multidrug efflux complex from *E. coli* are the aminoquinoline NSC33353 and the dihydroimidazoline NSC60339.

RND-type drug efflux transporters from the HAE-1 family confer resistance to a variety of structurally diverse compounds. *E. coli* AcrB exhibits a broad substrate specificity, including different classes of antibiotics, like macrolides, fluoroquinolones, β-lactams, tetracyclines, chloramphenicol (CAM), rifampicin (RIF), NOV, but not aminoglycosides. Further substrates are dyes such as acriflavine, ETH, simple solvents such as hexane, bile salts such as taurocholate, and detergents like Triton-X-100, DDM, and SDS [121, 122] (Fig. 6). As a common feature, those compounds are of rather hydrophobic or amphiphilic nature [13, 121]. The export of aminoglycosides like gentamycin and dianionic β-lactams such as aztreonam or carbenicillin is in *E. coli* assigned to AcrD [20, 89, 91, 123]. In *P. aeruginosa*, aminoglycoside resistance is attributed to MexY activity, but not MexB [124, 125]. On the other hand, in *A. baumannii* the RND efflux pump AdeB was reported to exhibit more diverse substrate specificity compared to AcrB or MexB, and its substrates includes aminoglycosides and dianionic β-lactams [126–131].

In the past decades, extensive efforts have been undertaken to shed light onto the molecular basis of multidrug recognition by RND-type transporters [60]. Elkins and Nikaido used chimeric constructs between AcrB and AcrD to show that the substrate specificity of RND transporters is predominantly determined within the periplasmic part [28]. Similar observations were made with chimaeras of AcrB/MexB [29] and MexB/MexY [30].

### Drug binding to the access pocket

**Fig. 7.**
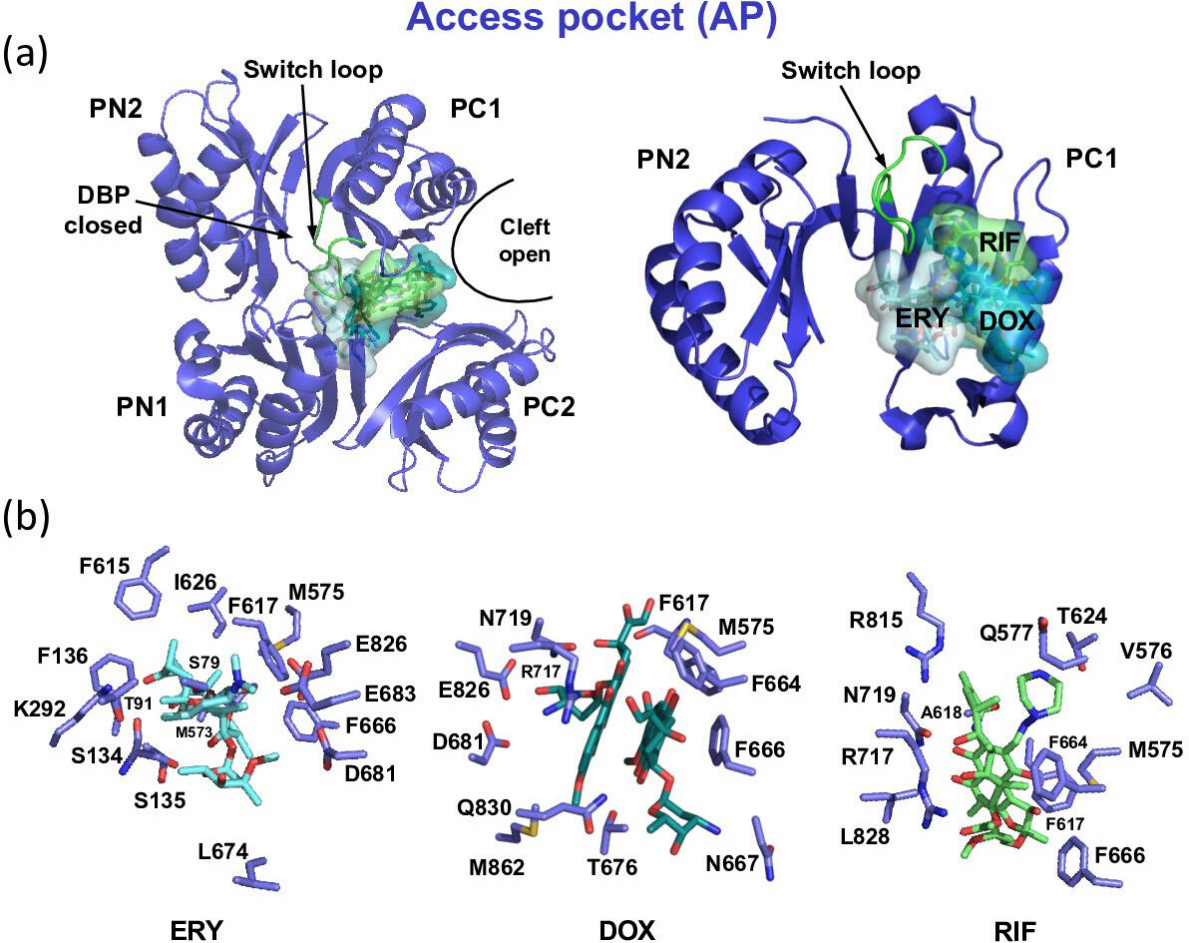
Substrate binding to the access pocket (AP) of the AcrB L protomer. (a) Top view (left) and side view (right) onto the porter domain comprising the PN1/PN2 and PC1/PC2 subdomains of the L protomer (blue cartoon). The PN1 and PC2 subdomains are omitted in the image on the right for a clearer view on the substrate binding. Erythromycin (ERY, pale cyan colour), doxorubicin dimer (DOX, teal) and rifampicin (RIF, green) bind within the access pocket (AP), substrates are depicted in sticks and surface representation. The switch loop is highlighted in green. (b) From left to right: Interaction sites of ERY (palecyan) (PDB ID: 3AOB), DOX dimer (teal) (PDB ID: 4DX7) and RIF (green) (PDB ID: 3ABC). AcrB residues involved in substrate binding are shown in blue sticks.

**Fig. 8.**
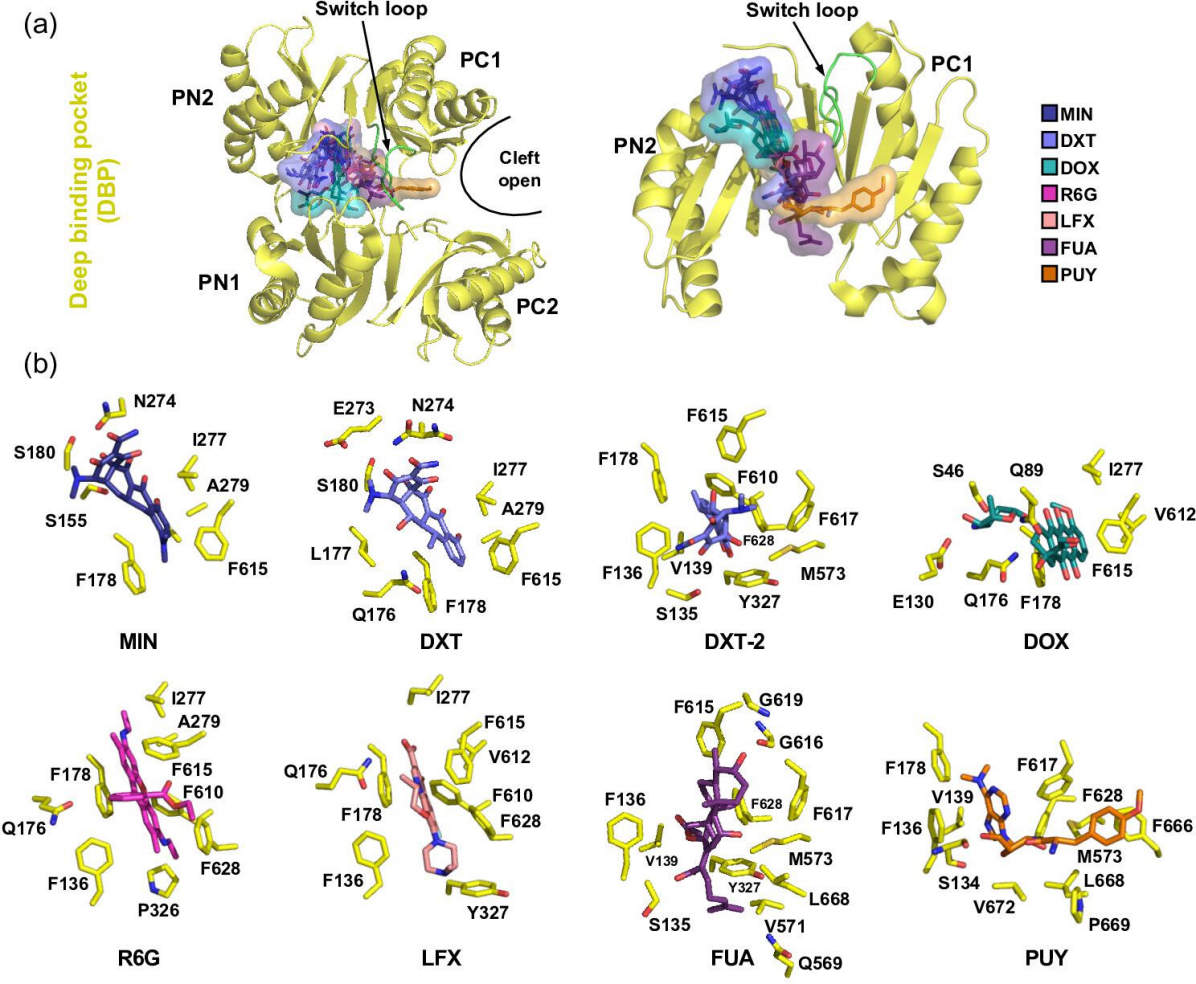
Substrate interactions sites within the AcrB deep binding pocket (DBP). (**a**) Top view (left) and side view (right) onto the porter domain (with its PN1/PN2 and PC1/PC2 subdomains) of the T protomer (yellow cartoon). The PN1 and PC2 subdomains are omitted in the image on the right for a clearer view on the substrate binding. Binding of AcrB substrates within the DPB is depicted in sticks and surface representation. The superimposition of the substrate coordinates from the AcrB/substrate co-structures illustrates how each substrate binds to a distinct site within the large DBP but interacts with partially shared sets of AcrB residues. The switch loop is highlighted in green. (**b**) From left to right: Substrate interaction sites of minocycline (MIN, dark blue colour, PDB ID: 4DX5), doxycycline (DXT, pale blue, PDB ID: 7B8R), doxorubicin (DOX, teal, PDB ID: 4DX7), rhodamine 6G (R6G, pink, PDB ID: 5ENS), levofloxacin (LFX, salmon, PDB ID: 7B8R), fusidic acid (FUA, violet, PDB ID: 7B8S) and puromycin (PUY, orange, PDB ID: 5NC5) are shown.

Early structures of symmetric AcrB (i.e. in the LLL state) were reported to have substrates bound to the inner wall of the trimeric TMD cavity [132–134], whereas none of the asymmetric structures at higher resolution displayed any of the substrates bound within the central cavity [102, 105, 106], with the exception of the recent cryo-EM co-structure of AcrD in complex with gentamycin [83]. Electron densities from co-crystals of symmetric AcrB indicated substrates bound to the open PC1/PC2 cleft, proximal to the AP [133, 135]. It has been suggested that the latter observation might represent an earlier step in substrate uptake by the unoccupied resting LLL state [106]. For asymmetric AcrB in complex with high molecular mass drugs, structures revealed binding of RIF, 3-formylrifamycin SV (3-FOR), dimeric doxorubicin (DOX) or erythromycin (ERY) to the AP of the L protomer [105, 106, 116] (Fig. 7). The binding of dimeric DOX to the AP in the L protomer may represent a preliminary stage to the binding of the monomeric ligand to the DBP in the T conformation [106] (Fig. 8) and might be seen as an example of how the decision to finally extrude a compound is made in several successive steps (multistage recognition). While RIF appears to be stalled proximal to the switch loop, ERY was found in a location below the switch loop [105]. This appears similar to the position of puromycin (PUY) in the T protomer [37], and both might represent states close to the conformational L to T transition (Figs 7 and 8).

### Drug binding to the deep binding pocket

The DBP binding modes of substrates co-crystallized with AcrB to date is shown in Fig. 8 [78, 102, 105, 106 and 136]. Superimposition of these ligand coordinates (Fig. 8a) illustrates that the different substrate classes share partially overlapping binding sites within the regions of the entire DBP. Hence, AcrB does not only feature multiple binding pockets, but additional ‘multifunctional sites’ within those pockets, where subsets of amino acid side chains enable the binding of aromatic, hydrophobic and polar groups [105, 136–138]. Indeed, MD simulations identified five such multifunctional sites within the AcrB PD [90], highlighting the enormous plasticity of the AcrB DBP. Moreover, in the same study it was highlighted that recognition via a simple steric filter, as for example molecular weight or minimal projection area of the drugs, appears unlikely, as both AP and DBP have volumes and shapes at least twice as voluminous as the largest known AcrB substrate, bleomycin [90]. Compared to the AP, the DBP is clearly more lipophilic and was suggested to act as ‘lipophilicity-based selectivity’ filter for low molecular mass drugs [90]. In a docking study to address this potential drug binding flexibility, substrates were grouped into ‘groove binders’, i.e. substrates binding to a narrow groove in the upper part of the DBP and ‘cave binders’, i.e. binding to a lower and wider part of the DBP. Substrates grouped as ‘mixed binders’, i.e. binding in between the groove and cave regions were likewise assigned [139, 140]. For each drug molecule, several binding sites within the DBP were identified, leading to the proposal of drug transport via a ‘multidrug oscillation mechanism’ [122]. According to the latter, drug molecules oscillate through the voluminous binding pocket, interacting with several transient binding sites. In this way, while the apparent drug binding affinity to each of the sites might be low, the total drug binding efficiency might be higher [137].

Binding of the low molecular mass drugs such as minocycline (MIN), DOX, rhodamine 6G (R6G), PUY [37, 102, 106, 141], DXT (doxycycline, a tetracycline), LFX (levofloxacin, a fluoroquinolone) and FUA (a fusidane) [78] was observed to the DBP of T protomer (Fig. 8). Unsurprisingly, the two tetracycline antibiotics MIN and DXT, that both comprise the typical tetracycline core but carry distinct substitutions at the C5, C6 and C7 positions, appear to bind the DBP in a highly congruent manner (Fig. 8a). The carboxy amide group of MIN/DXT interacts with the N274 polar side chain, while F178 and F615 sandwich the tetracycline aromatic d-ring (Fig. 8b). Furthermore, MIN and DXT are engaged in a water-mediated H-bond network, extending from the MIN/DXT 12a-hydroxyl group. The DXT co-structure revealed another, hitherto unknown, binding site (DXT-2) for tetracycline antibiotics within the DBP cave (Fig. 8a), where DXT-2 is almost exclusively involved in hydrophobic interactions with the hydrophobic trap (HT) Phe-cluster (F136, F178, F610, F628) and Y327 (Fig. 8b). The DXT-2 binding site is anticipated to depict a lower affinity binding site, as reflected by its higher B-factor. The two DXT binding sites might thus represent snapshots of the tetracycline compound along its transport pathway through the AcrB PD [78]. A similar conclusion was made for ETH binding in the AdeB component of the AdeABC efflux pump from *A. baumannii*. In the different T states of the solved AdeB asymmetric structures, two or three ETH molecules were bound to the DBP or to the entry cleft of the AP. Also here the conclusion was made that the different binding sites might reflect different binding sites of the drug along the drug transport pathway *in vivo* [77, 142]. Compared to the multiple site drug binding of DXT, the fluoroquinolone LFX displays a distinct single binding site in *E. coli* AcrB (Fig. 8b). Superimposition of LFX coordinates with those of other co-crystallized AcrB substrates revealed that the LFX binding site within the DBP and HT substantially overlaps with the binding site previously reported for the dye R6G [141] (Fig. 8b). Both compounds greatly differ in their physicochemical properties (Table 1), with LFX carrying a localized negative charge and a single aromatic ring, while R6G carries a delocalized positive charge and is of polyaromatic nature. Despite their obvious physicochemical differences, the AcrBper/LFX co-structure shows LFX binding at the same planar level as R6G, albeit slightly shifted toward Y327 in the lower (entrance) part of the DBP region. Both molecules mainly interact with the F178 and F628 side chains via π-π-stacking, complexing the quinolin-4-one core of LFX and 2,7-dimethylxanthene core of R6G, respectively. In contrast, the fusidane FUA binds to a more proximal part of the AcrB DBP, where the drug molecule is involved in various interactions with AcrB side chains of the DBP cave and AP/DBP interface (Fig. 8). The FUA 3-hydroxyl group forms a H-bond to the switch loop with the G616 main chain, while the FUA carboxylic acid group is engaged in a water-mediated H-bond network with the main chain of F136. Once the drugs are tightly bound in the T protomer, energy-dependent T to O transition results in the closure of the periplasmic binding pockets and concomitant opening of the EG within the O protomer and hence efflux of the compounds. For AcrB, both the existence of multiple substrate entry sites [102–105, 113–116] as well as the variable nature of the two large periplasmic multifunctional substrate AP and DBP [102, 105, 106], contribute to the broad substrate polyspecificity of this RND-type efflux pump [78].

**Table 1.** Physicochemical properties of co-crystallized AcrB substrates

Binding mode	AP		DBP						
**Compound**	**RIF**	**ERY**	**MIN**	**DXT**	**DOX**	**R6G**	**LFX**	**FUA**	**PUY**
**Charge (pH 7.4)**	−0.49	0.98	−0.39	−0.63	0.79	1	−0.8	−1	0.81
**HDB count**	6	5	5	6	6	2	1	3	4
**HBA count**	14	13	9	9	12	3	7	5	10
**Aromatic ring count**	2	0	1	1	2	4	2	0	3
**logP**	2.95	2.60	−2.57	−3.65	0.54	5.35	0.09	4.42	−0.30
**MPA (Å^2^)**	121.4	107.2	63.8	66.4	76.1	79.9	45.7	79.6	67.1
**MW (g mol^−1^)**	823	734	457	444	544	444	361	517	472

Compounds RIF and ERY bind to the AcrB access pocket (AP), whereas the other compounds were found inside the deep binding pocket (DBP). The listed properties of the compounds are their charge at 7.4, the number of hydrogen bond donors (HBD) and hydrogen bond acceptors (HBA), the number of aromatic rings, partition coefficient (logP), minimal protection area (MPA) and molecular weight (MW). Data was calculated using Chemicalize.org by ChemAxon. RIF: rifampicin, ERY: erythromycin, MIN: minocycline, DXT: doxycycline, DOX: doxorubicin, R6G: rhodamine 6G, LFX: levofloxacin, FUA: fusidic acid, PUY: puromycin.

### The role of the switch loop in drug transport

Whereas the presence of CH3 and CH4 imply that drugs can be sequestered by the T protomer directly into the DBP, CH1 and CH2 entry, i.e. the initial binding step of drugs to the TMH8/TMH9 groove (CH1) or the AP (CH2) may rather occur in the L protomer. Nevertheless, as CH1 and CH2 are also present in the T protomer and binding of drugs is also observed at the TMH8/TMH9 groove (CH1 entry) and CH2/AP (albeit in a G619P switch loop variant) in the T protomer [116], there is still lack of understanding of the sequential steps of drug transport. For AcrB wild-type, the substrate DDM most likely enters CH1 after being caught in the TMH7/8 groove in the L state. Co-crystal structures of bound DDM to the TMH7/8 groove in the L protomer most likely indicate this initial state of binding [104, 106]. The ligand subsequently moves into the entrance of CH1 [143] and finally unlocks the TM8/PC2 pathway where DDM is bound proximal to the AP and DBP near the switch loop. In this conformation, which is still the L state with a closed DBP, the c-loop adjusts its conformation substantially to stabilize the ligand binding and TMH8 is partially tilted as it is in the T state [116]. For larger drugs such as the macrolide ERY, sequesteration to the L protomer AP via CH2 entry [105], is followed by an L to T protomer conformational change as the drug moves from the AP into the DBP. The L to T transition includes conformational change of the switch loop and the PN2/PC1 subdomains. Resistance against ERY was shown to be drastically reduced in cells harbouring the switch loop variant G616N, most probably due to induced rigidity of the loop motif, indicating that switch loop flexibility indeed is important for substrate recognition/specificity and/or transport [144, 145]. The switch loop was suggested to be essential for drug efflux, as replacement of the region G614-N623 of this loop by a short double alanine linker caused complete loss of function [145], but not for drug binding [143]. According to another study, a slightly different deletion of the AcrB switch loop, i.e. F615G-Δ[G616-R620] does not affect susceptibilities toward DOX, suggesting that the loop is not required for the export of this particular drug [146]. A first analysis by introduction of Gly to Pro substitutions at positions 616 and 619 in the switch loop suggests a total loss of transport activity [105, 145]. However, additional substitution of the large side chains F615 and F617 to Ala within the G616P/G619P switch loop resulted in the recovery of activity! It appears therefore that the F615 and F617 side chains block drug transport toward the DBP when the switch loop is rigidified, but the rigidity of the loop itself is not responsible for loss of transport, as the rigid loop with A615/A617 instead of F615/F617 shows ample efflux activity [145].

## Comparison between *A. baumannii* AdeB and *E. coli* AcrB

### Structural comparison of *A. baumannii* AdeB and *E. coli* AcrB

**Fig. 9.**
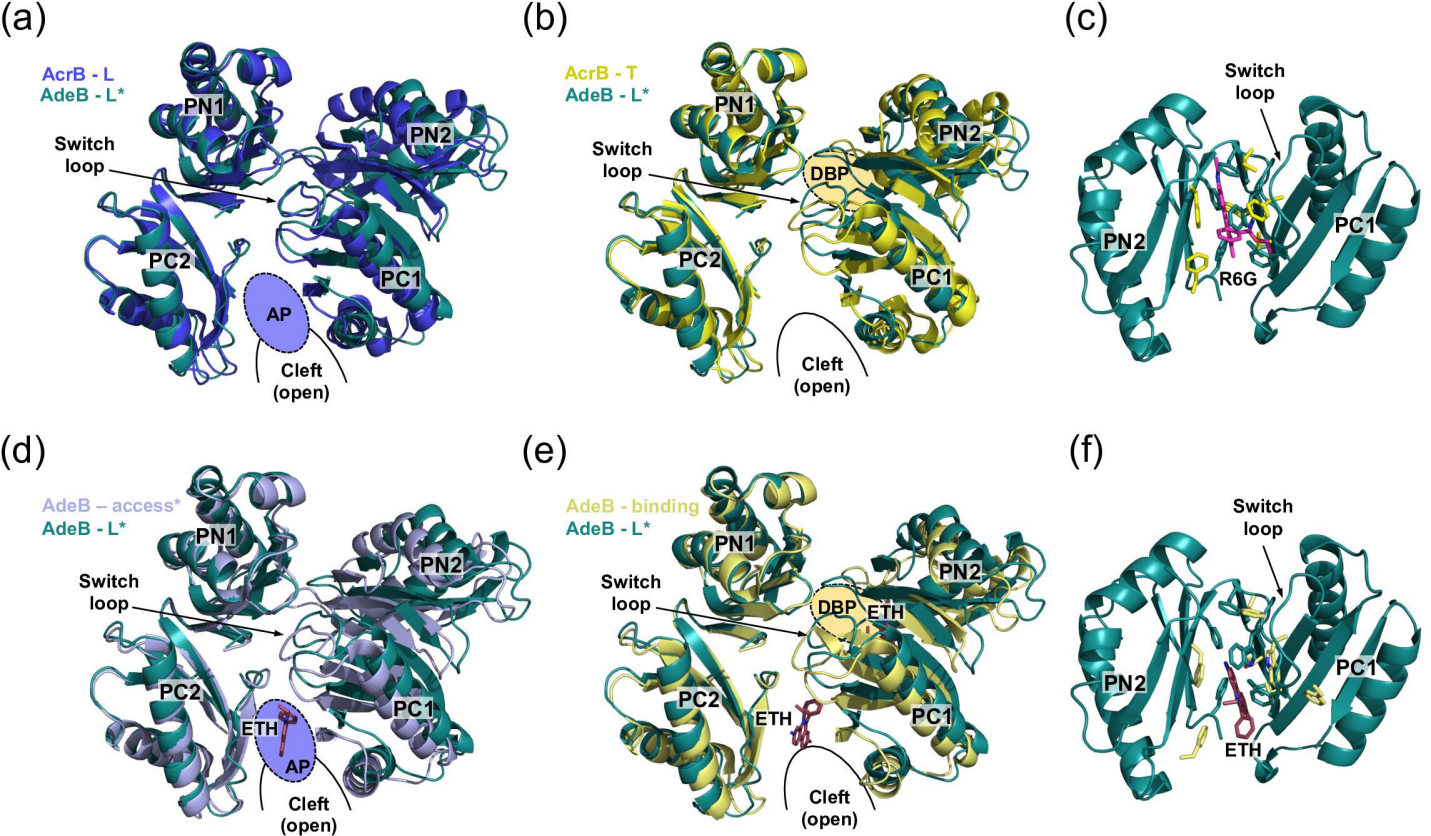
Comparison of AdeB L* conformation with the L/access and T/binding conformation of AcrB or AdeB. (**a**) Top view on the periplasmic porter domain (PD) of AdeB L* conformer superimposed on the PD of the AcrB L conformer, (**b**) AcrB T conformer, (**d**) AdeB access conformer, or (**e**) AdeB binding conformer. (**c**) Side view on the PN2 and PC1 subdomains of the AdeB L* conformer superimposed on (**c**) AcrB T conformer, or (**f**) AdeB binding conformer. For the latter two, only the drug (R6G, ETH) interacting residue side chains are shown as sticks in yellow colour. The same residues have a different orientation in the AdeB L* conformer (residues shown in teal-coloured sticks) and do not allow for ligand binding. R6G is shown in pink sticks (**c**) and ETH (**d–f**) in berry sticks. The PD subdomains of AdeB L* (in teal colour, PDB ID: 7B8Q), AcrB L (blue, PDB ID: 4D5), AcrB T (yellow, 5ENS), AdeB access (pale blue, PDB ID: 7 KGI), and AdeB binding (pale yellow, PDB ID: 7 KGI) are represented as cartoon. The architecture of the AdeB L* state shows the highest resemblance with the AcrB T state (shown in b). However, the PN2/PC1 subdomains adopt distinct conformations (**b, e**) and hence different side chain conformations (**c, f**). As a consequence, the DBP is closed in AdeB L*.

Recent single-particle cryo-EM structures of *A. baumannii* AdeB revealed a hitherto unknow trimer conformation, L*OO. Here, the L* protomer represents an intermediate state during L to T transition [78] (Fig. 9). Although the overall architecture of AdeB L* rather resembles the AcrB T-state, the DBP that is flanked by the PN2/PC1 and PN1 subdomains, appears to be closed in L*. The closed DBP appearance is due to distinct conformations of the PN2 (quite different from both AcrB L and T states) and PC1 (more like the AcrB L state) subdomains (Fig. 9). Three entry tunnels, corresponding to channels CH1, CH2, and CH3 in AcrB [102–105, 113], were identified in the L* protomer that are directed toward the closed DBP. In another study, formation of asymmetric AdeB LTO, TOO, as well as RTO (R=resting) assemblies were observed only in the presence of the drug substrate ETH [77, 142]. The R state, originally described as the resting state of apo HME transporter CusA from *E. coli* [147, 148], was also observed for the *C. jejuni* HAE-1-type pump CmeB and was proposed to represent one of the intermediate states the transporter has to adopt during the transport cycle [75]. In this R state, the periplasmic cleft, and all channels, including the exit tunnel and EG, are closed. Based on the CmeB X-ray structures and functional dynamics studies via single-molecule fluorescence resonance energy transfer (sm-FRET) with CmeB reconstituted in liposomes, a transport mechanism was proposed in which the CmeB protomers function independently within the trimer [75]. Whether concerted cycling rather than the independent cycling of the protomers is also dependent on the tripartite assembly such as in this case CmeABC [42], is an open question and needs further experimentation. It appears therefore, that the transport cycle of RND-type efflux pumps might be more complex as anticipated by the initial functional rotation hypothesis [102, 103] and this hypothesis might not necessarily be for all multidrug pumping RND systems an accurate description of the transport cycle.

### Comparison of drug binding in *A. baumannii* AdeB and *E. coli* AcrB

In a recent comparative mutagenesis study [78], the drug efflux capacities of *A. baumannii* AdeB DBP variants were investigated via drug agar plate dilution assays and compared to those of wild-type AdeB. Based on the known substrate binding modes to the homolog RND transporter AcrB from *E. coli*, 20 AdeB DBP variants were generated, in which the selected residues were either mutated to alanine or to the corresponding residue in AcrB.

**Fig. 10.**
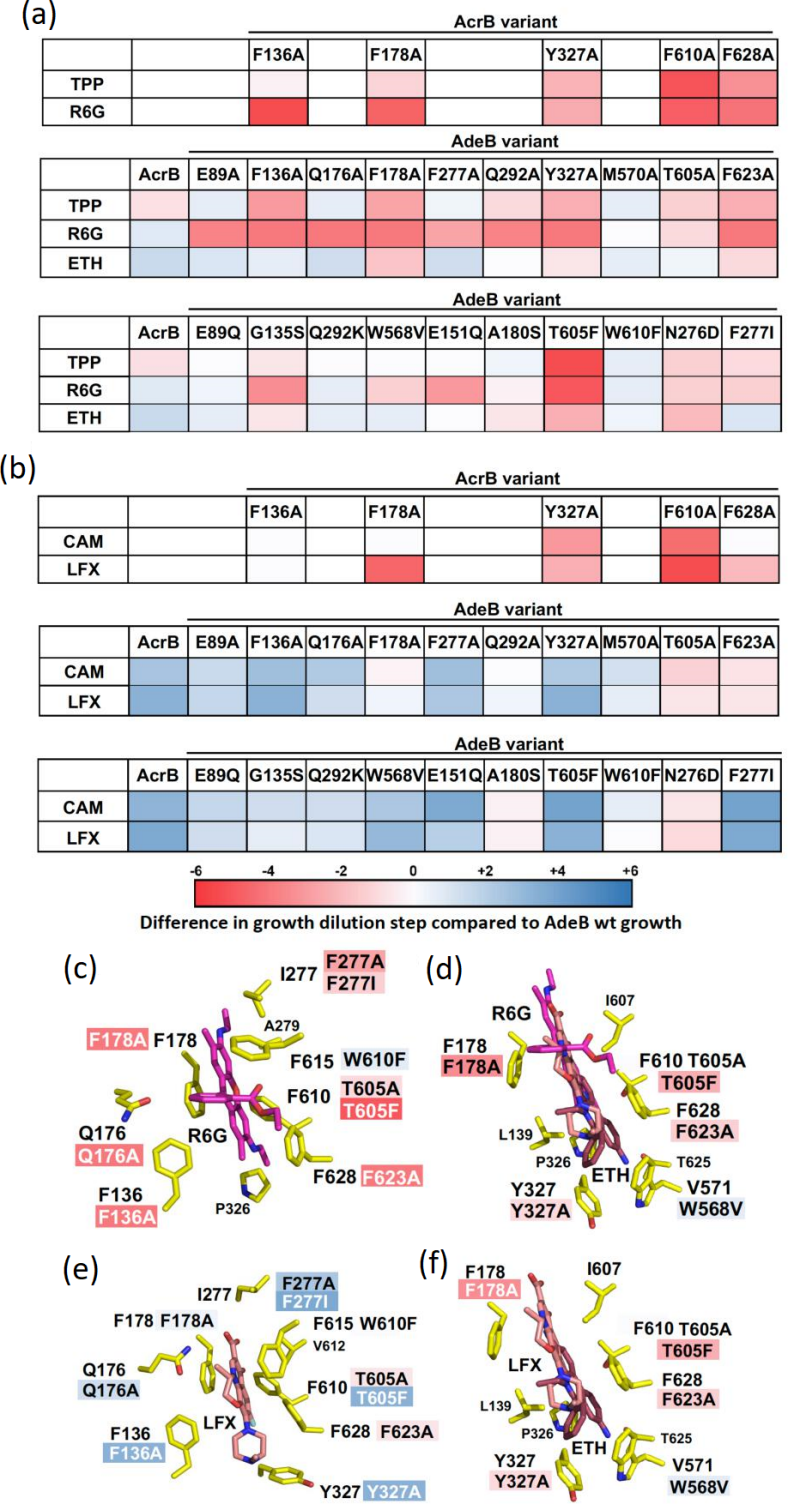
Activity analysis relative to AdeB wild-type activity and comparison of drug DBP binding modes in AdeB and AcrB for TPP, R6G, ETH, CAM and LFX. (**a, b**) Analysis of drug agar plate dilution assays with AcrB wt and indicated AcrB DBP Ala-variants (top panel), AdeB wt and AdeB DBP Ala-variants (middle panel) or DBP AdeB-to-AcrB variants (bottom panel) for (**a**) TPP, R6G and ETH or (**b**) CAM and LFX. The activity of each variant is colour coded from blue (+6) (hyperactive variants, i.e. more active than AdeB wt) to white (0) (variant activity equal to AdeB wt) to red (−6) (variants with lower activity than AdeB wt). Results were analysed by counting the number of dilution steps showing cell growth. For that, 10^0^-10^−5^ dilutions from a culture with OD_600_=1 were spotted on LB + drug agar plates. Values from three independent biological repeats were averaged and normalized by subtraction of the negative control (AcrB D407N) and the number of dilution steps showing cell growth for wild-type AcrB or AdeB transporter. (**c, e**) Substrate interaction sites of (**c**) rhodamine 6G (R6G, in pink-coloured sticks) (PDB ID: 5ENS) and (**e**) levofloxacin (LFX, in salmon-coloured sticks) (PDB ID: 7B8R) within the AcrB DBP. (**d, f**) Superimposition of the substrate interaction site of ethidium (ETH, in berry-coloured sticks) (PDB ID: 7 KGI) within the AdeB DBP superimposed with (**d**) rhodamine 6G (R6G, in pink-coloured sticks) (PDB ID: 5ENS) and superimposed with (**f**) levofloxacin (LFX, in salmon-coloured sticks) (PDB ID: 7B8R). The side chains shown are from the ETH-bound AdeB co-structure (PDB ID: 7 KGI), and the coordinates of R6G and LFX are from the AcrB co-structures. The number and one-letter code of the amino acid side chains (in plain, black-coloured font) are from the AcrB co-structures, whereas the substitution variants indicated use the one-letter code and numbering from AdeB. Protein residues involved in ligand binding are shown as sticks (carbon=yellow; nitrogen=blue; oxygen=red; sulphur=gold). Susceptibility effects upon amino acid substitution within the AdeB DBP are indicated in blue or red shadings on the residue number and single letter amino acid code (in black or white coloured font). Whereas most substitutions result in growth reduction on R6G (**c**) and ETH (**d, f**), a majority of substitutions cause better than AdeB wild-type growth on LFX (**e**), despite its similar binding mode compared to ETH in the DBP (**f**).

**Fig. 11.**
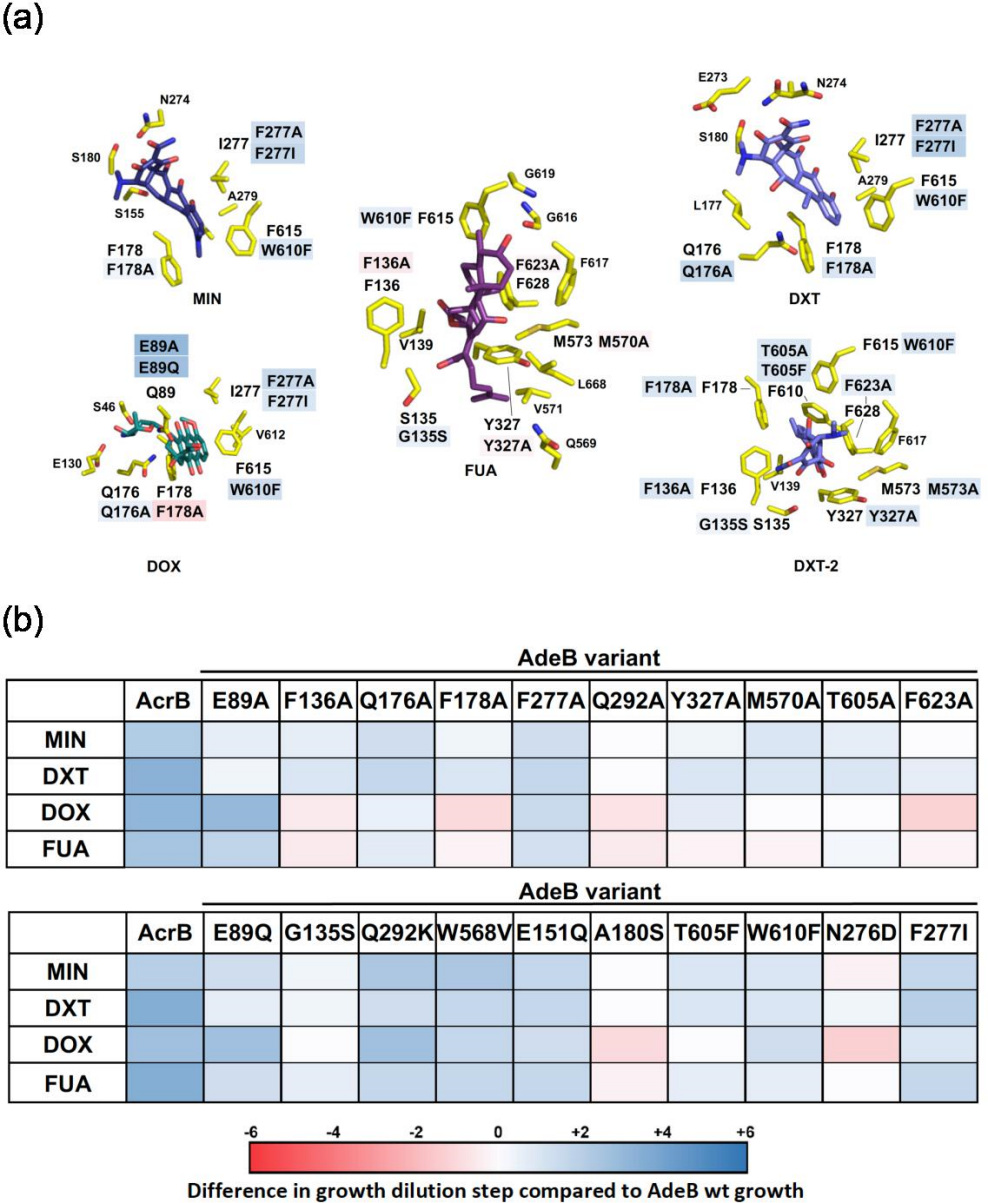
Activity analysis relative to AdeB wild-type activity and comparison of drug DBP binding modes in AdeB and AcrB for MIN, DXT, DOX, and FUA. (**a**) Substrate interaction sites within the AcrB DBP of minocycline (MIN, dark blue colour, PDB ID: 4DX5), doxycycline (DXT/DXT-2, pale blue, PDB ID: 7B8R), doxorubicin (DOX, teal, PDB ID: 4DX7) and fusidic acid (FUA, violet, PDB ID: 7B8S). AcrB residues (one-letter code and position number) involved in ligand binding are shown as sticks (carbon=yellow; nitrogen=blue; oxygen=red; sulphur=gold). (**b**) Susceptibility effects upon amino acid substitution within the AdeB DBP are indicated in blue or red shadings on the residue indicated: blue (+6) (hyperactive variants) to white (0) (variant activity equal to AdeB wt) to red (−6) (less-active variants than AdeB wt). Specifics of the activity analysis are mentioned in the legend of Fig. 10.

Overall, the efflux pump variant susceptibility data emphasize the broad substrate spectrum of AdeB, as all tested AcrB substrates, R6G and ETH (dyes), TPP (tetraphenylphosphonium), DOX (anthracycline), MIN and DXT (tetracyclines), LFX (fluoroquinolone), CAM (phenicol) and FUA (fusidane), were shown to be also transported by the wild-type AdeB protein. However, drug efflux capacities of these pumps when expressed in *E. coli* greatly varied among the tested compound classes. Compared to AcrB, AdeB appears to confer much lower resistance levels toward all non- and weakly-polyaromatic drugs, like FUA (non-aromatic), MIN, DXT, LFX and CAM (all have one aromatic ring), and DOX (two aromatic rings), suggesting that these are rather weak AdeB substrates (Figs 10 and 11). In contrast, compounds featuring three or more aromatic rings, i.e. R6G, ETH, and TPP, were efficiently transported by AdeB, as wild-type *adeB* expressing *E. coli* displayed only slightly lower (ETH), equal (R6G) or even higher (TPP) cell growth compared to *acrB* expressing cells (Fig. 10).

A set of AdeB cryo-EM structures in complex with ETH at resolutions between 3.0–3.8 Å was recently reported by Morgan *et al.* [77]. The co-structures revealed three distinct ETH interaction sites within the AdeB. One binding site was at the periplasmic cleft, another at the DBP, and a third at the HT region (Fig. 9). Binding of ETH within the DBP mainly involves an extensive π-π-stacking interaction of the ETH phenanthridine core with the F623 aromatic side chain. Similarly, the ETH molecule bound to the DBP HT mainly interacts with the F178 and W610 aromatic side chains. Further hydrophobic interactions with F136, F277, Y327, M570 and T605 were observed to contribute to ETH binding in AdeB. In agreement with these structural data, mutagenesis data by Ornik-Cha, Wilhelm *et al.* [78] (Fig. 10) showed the increase of ETH susceptibility on cells harbouring the AdeB F178A and F623A variants, most likely due to the lacking π-π-stacking interactions with the ETH phenanthridine core observed in the cryo-EM co-structure. Other substitutions resulted in increasing resistance toward ETH (Fig. 10a, b).

In a recent publication, Leus *et al.* [149] investigated the three ETH binding sites of AdeB via determination of the MICs and IC_50_ of different antibiotics in *A. baumannii* cells expressing mutated *adeB* genes, resulting in cells producing AdeB substitution variants. They found that functional interactions between the structurally identified binding sites are non-additive, meaning that different combinations of side chain substitutions in the various drug binding sites result in different antibiotic susceptibility effects. The highly conserved F178, when replaced with Cys in the F178C mutant, leads to a predictable decrease in ETH resistance, but also to an unexpected increase in gentamicin and zeocin resistance. The loss of affinity for ETH can be explained by the removal of the phenyl ring as tight interaction partner. The Cys substitution also creates a less hydrophobic environment for more hydrophilic drugs like gentamycin and zeocin. Zeocin, a very large drug, may benefit from the removal of a bulky side chain, potentially providing additional space. This idea of a larger space for accommodating large hydrophilic drugs is supported by the observation that the AdeB F178A variant also provides zeocin hyperresistance to *A. baumannii* cells. When the F178C substitution is combined with F277C or W610C located in the same binding pocket, AdeB loses its resistance to all tested antibiotics and even makes the cells more susceptible than the efflux-deficient controls. Leus *et al.* [149] suggest that this might be due to the cross-linking between the Cys residues, stalling the protomers in a transport inactive state and even facilitating the influx of drugs into the cells. The combination of F178A and F277C, as a control to address the effect of potential cross-linking vs. the effect of the change of physicochemical characteristic due to the substitution, indeed showed for this combination that zeocin was again better transported and caused zeocin hyperresistance, whereas gentamicin showed wild-type-like IC_50_ values. Since zeocin is a large compound, Leus *et al.* [149] anticipated that this drug might be initially binding to the access pocket, as all larger substrates such as RIF and macrolides have been shown to be bound there [105, 116, 143]. Indeed, the F178C deep binding pocket substitution, causing hyperresistance toward zeocin, combined with an access pocket substitution (D664C or W708C), led to a complete loss of zeocin hyperresistance and even led to lower resistance compared to cells expressing wild-type AdeB. Ornik-Cha, Wilhelm *et al.* [78] also observed the hyperresistance phenotypes for AdeB variants with DBP substitutions. This was especially toward non- and weakly-aromatic drugs. The phenotypes were often observed when single-side chains in AdeB were replaced by AcrB residues at their homolog positions (See Fig. 10e for LFX, and Fig. 11a for MIN, DXT, DOX and FUA).

### Comparison of AdeB and AcrB variant resistance phenotypes

The residues within the AcrB DBP and the residues from the HT Phe-cluster were shown to be involved in substrate and EPI binding (Fig. 8) [140, 141, 150, 151]. The two hydrophobic clusters are conserved among homologue RND transporters. For AcrB, one substitution, F610A, conferred increased susceptibilities toward almost all substrates tested, including MIN and DOX [152], as well as ETH, LFX, CAM [78, 152] and R6G [78]. MD simulations on DOX transport in the F610A variant suggested longer dwelling times of the drug molecule within the substituted binding pocked due to stronger protein-ligand interactions as compared to the wild-type transporter [153]. In a study by Bohnert *et al.* [152]*,* the MICs of a set of macrolide antibiotics, including ERY, were significantly decreased for the F136A, F178A and F628A variants, whereas resistance levels toward MIN and DOX, as well as ETH, LFX and CAM were not affected. Based on their studies, the authors suggested that the large number of Phe residues within the DBP and HT introduces a certain redundancy of aromatic side chains that would – upon a single Ala-substitution of a Phe – lead to only to slight substrate reorientation within the binding pocket, without generally compromising transport [152]. Impaired efflux capacities on TPP, R6G, CAM, and LFX were observed by Ornik-Cha, Wilhelm *et al.* for the AcrB F136A, F178A, Y327A, F610A, and F628A variants in drug agar plate dilution assays [78], but with clear differences between these variants (Fig. 10). The Ala-variants all conferred reduced resistance against R6G and TPP, albeit the latter to lesser extent for F136A and F178A. For CAM, only Y327A and F610A affected resistance. LFX resistance was compromised with all but the F136A variant. Differences between the two studies [78, 152] were thus seen in a clear loss of resistance for F178A and F628A against LFX in the plate dilution assay [78], whereas the MICs for LFX were not changed for these AcrB variants [152]. For CAM, the data was comparable between these two studies. Yao *et al.* [154] reported increased susceptibilities for the Y327A variant toward R6G, TPP and CAM, amongst other drugs (LFX was not tested). The only drug susceptibility unaffected by this substitution was for ETH. Here, data was consistent with the results obtained via the plate dilution assays [78] (Fig. 10a).

For AdeB, variants with single Ala-substitutions in the DBP displayed a strong phenotype distinct from wild-type AdeB. Especially the removal of a single aromatic Phe or Tyr side chain within the HT (F136A, F178A, F277A, Y327A, F623A) led to loss of resistance toward TPP (except for F277A), R6G and ETH (except for F136A and F277A) [78]. The effects were less pronounced for ETH. Resistance toward LFX and CAM was, in contrast, greatly enhanced by the substitutions, except for F178A and F623A (Fig. 10). This increase of resistance (i.e. hyperresistance, better than wild-type resistance) by single substitutions was also reported by Leus *et al.* [149]. The hyperresistance phenotype is in addition strongly present with other AdeB substrates like MIN and DXT (Fig. 11). Resistance against DOX and FUA was in addition to increased also mildly decreased for some substitutions like F136A, F178A, and F623A (Fig. 11). It appears that polyaromatic substrates such as TPP, R6G, ETH, DOX, and the very hydrophobic compound FUA, were more prone to Phe-to-Ala substitutions leading to a decrease of resistance, whereas more hydrophilic compounds such as CAM, LFX, MIN and DXT showed in most tested variants hyperresistance phenotypes [78] (Figs 10 and 11). To align these observations with the structural information available for AdeB [77, 78], not only the local properties within the drug binding sites have to be considered, but also the observed conformational states within the AdeB trimer, which are discussed in the next paragraph.

### Substrate binding to AdeB might occur via induced fit

In the absence of drug substrates, trimeric AdeB predominantly adopts a symmetric OOO conformation, in which all the protomers are in the O-state [76, 78]. In one study, however, an apo AdeB trimer structure determined by single-particle cryoEM adopted a hitherto unknown asymmetric conformation, designated L*OO [78]. In the presence of the AdeB substrate ETH, on the other hand, formation of asymmetric AdeB LTO (AdeB-III-Et), TOO (AdeB-I-Et), as well as RTO (R=resting, all channels closed) (AdeB-II-Et) assemblies were observed [77]. It appears therefore, that in absence of drugs, the OOO and the L*OO states are adopted, with the protomer in the L* conformation as initial entry for drugs. Once drugs are consistently present, structures comprising the LTO, TTO and RTO states are observed, and those structures display binding of one or two ETH molecules to the open DBP of the T protomer, while an additional ETH molecule is apparent in the AP of the same protomer (Fig. 9e). This contrasts with the trimeric assembly of AcrB, where the DBP presents itself open not only in the presence, but also in absence of substrates [101–104, 106]. Therefore, substrate binding in AcrB may rather occur by conformational selection, where incoming drugs permutate through numerous binding sites within the open substrate binding pocket [122].

Based on these findings, the AdeB transport cycle is postulated to start with drug binding to the L* protomer within the L*OO trimer [142]. This binding event to the AP might induce the DBP to open and adopt its binding capacity to the incoming drug, akin to an induced fit mechanism. Consequently, drug binding is envisioned to trigger L* to T transition, possibly leading to the TOO (AdeB-I-Et) and then LTO (AdeB-III-Et) conformations reported by Morgan *et al.* [77]. Subsequent transport cycles might follow the mechanism proposed for *E. coli* AcrB [63, 96, 102–104, 111, 142]. The tight arrangement of hydrophobic side chains within the AdeB DBP Phe-cluster of L* (Fig. 9c, f) might present a more suitable environment for polyaromatic compounds, as they presumably easily adopt to the incoming drug, allowing tight interactions between the aromatic entities of both substrate and AdeB side chains. Although the binding pocket must be flexible enough to accommodate a variety of chemically diverse substrates, the pocket architecture of L* might be less suited for non-polyaromatic drugs like CAM, LFX, MIN and DXT, where the binding presumably involves a higher number of polar interactions. The enhanced efflux capacities observed for these drugs by AdeB variants upon single DPB Ala-substitutions of Phe, Tyr, or Trp residues, might be explained by a reduction of van der Waals interactions in the DBP and a widening of the binding site and/or a reduction of binding energy required to open the van der Waals-glued hydrophobic moieties via an induced fit [78]. This might enable the improved transport of (weaker) non-polyaromatic substrates in these variants. The AdeB variants with substitution changes into AcrB residues at homolog positions likewise improved the efflux capacity for these drugs (Fig. 11).

The comparison between two related RND-type efflux pumps AdeB and AcrB which show nearly identical drug substrate specificities indicates that the molecular determinants of drug binding and their molecular mechanism of transport might be more diverse than it might appear on first sight. When comparing the molecular properties of efflux pumps, the experimental setting must be carefully controlled, the experimental background (strains, growth medium, method of activity measurements) identical for all efflux pumps and their variants (see Teelucksing *et al.* [20]). Ideally, the study of efflux pumps and their variants would be tested in an *in vitro* setting, like in a proteoliposome drug efflux assay [155, 156], to exclude pleiotropic effects due to physiological adaptation in a living cell, as may occur during MIC and plate dilution assays. On the other hand, *in vitro* reconstitution into proteoliposomes necessitates purification of the drug efflux pumps, in case of the RND-type efflux pumps and elaborate setup including all three protein components (e.g. AcrA, AcrB, and TolC) which will prevent throughput analysis of the high number of variants needed for careful interpretation. Unless this challenge has been solved, analysis of multiple variants will be dependent on indirect activity measurements (susceptibility testing of bacteria expressing the pump genes), or measuring the direct efflux of fluorescent compounds [157] or by determination of drug uptake into cells [158–160].

## Efflux pump inhibitors

### Inhibitors acting against RND-type efflux pumps from *Enterobacteriaceae*

Given their central role in both intrinsic and acquired (multi) drug resistance [161], RND-type efflux systems are highly attractive drug targets. RND pump inhibition by small molecule EPIs was not only shown to restore the efficacy of antibiotics at low drug concentrations [162] but was also reported to decrease the emerge of resistance [163], to abolish biofilm formation [164] and to reduce the virulence of enteric pathogens [162, 165]. Thus, EPIs could be very powerful as adjunctive therapy in combination with (existing) antibiotics. However, the compound needs to cross the OM that constitutes a highly effective permeability barrier in Gram-negative bacteria. While penetration via the asymmetric OM is slow, uptake via porin channels requires the molecules to be small and rather hydrophilic. In contrast, the RND substrate binding pocket, that appears to be the target of most (potent) EPIs under development so far (see below), is of rather hydrophobic nature. Consequently, effective EPIs face often solubility challenges and display unfavourable pharmacokinetic and toxicological profiles [166, 167].

Back in the year 1999, Renau *et al.* [168] presented a family of peptidomimetics that constituted the first class of broad-spectrum EPIs active against various Gram-negative bacteria, including *P. aeruginosa* and *E. coli*. The lead compound phenylalanylarginine-β-naphthylamide (PAβN) (Fig. 6) was demonstrated to increase LFX susceptibility of *P. aeruginosa* by eight-fold (MPC_8_=10 µg ml^−1^, i.e. minimal potentiating concentration [MPC] at which the inhibitory activity of an antibiotic is potentiated eight-fold), and up to 64-fold in strains overexpressing the Mex efflux pumps [163, 168]. However, further development of PAβN analogues was discontinued due to nephrotoxicity [169, 170]. Another class of broad-spectrum EPIs, the arylpiperazines, including 1-(1-naphthylmethyl)-piperazine (NMP) [171] (Fig. 6). These are active against *E. coli* and other *Enterobacteriaceae* (such as *E. aerogenes* and *K. pneumoniae*) [171, 172]. However, high NMP concentrations are needed for EPI activity (LFX-MPC_8_=100 µg ml^−1^) [172]. Because of low potency and the fact that NMP might act as serotonin antagonist, the further development of this EPI series was halted [162]. Moreover, a series of pyridopyrimidine derivates was shown to specifically inhibit the MexAB-OprM pump system in *P. aeruginosa* [173]. The lead compound D13-9001 (Fig. 6), carrying a piperidine moiety with a quaternary ammonium salt side chain, displayed high potency (LFX-MPC_8_=2 µg ml^−1^) and good pharmacokinetic profiles [174]. However, as pyridopyrimidines show only low activity for *Enterobacteriaceae* and do not inhibit MexY [173], their further development has not been pursued. In 2014, Opperman *et al.* [175] from Microbiotix presented a novel and very promising EPI based on a pyranopyridine scaffold, MBX2319 (Fig. 6). Using a combination of checkerboard, time-kill, and efflux assays, they demonstrated its ability to increase the potency of a broad set of antibiotics (including fluoroquinolones and β-lactams) against *E. coli* by inhibiting drug efflux via the AcrAB-TolC system, without exhibiting any membrane-disrupting or antibacterial activity (MIC ≥100 µM) [175]. MBX2319 showed activity against other Gram-negative bacteria including *S. enterica*, *E. aerogenes*, *K. pneumoniae* and, albeit to a lesser extent, *P. aeruginosa* (most likely due to reduced OM permeability). Lead optimization by systematic variation of the substitutions around the pyranopyridine core revealed that the nitrile, dimethylenesulfide and gem-dimethyl groups are important for maintaining activity [176]. Changes at the morpholinyl moiety, however, led to improved microsomal stability and solubility, and the addition of non-acidic substituents to the phenyl group improved potency and *in vitro* pharmacokinetic properties [176]. Especially combination of 2,6-dimethylmorpholinyl and 4-acetamidophenyl (MBX3132) or 4-acrylamidophenyl (MBX3135) resulted in a substantial increase in potency (LFX-MPC_4_=0.1 µM) compared with MBX2319 (LFX-MPC_4_=3.1 µM) [176]. Hence, pyranopyridine-based EPIs are very promising candidates for further clinical development.

**Fig. 12.**
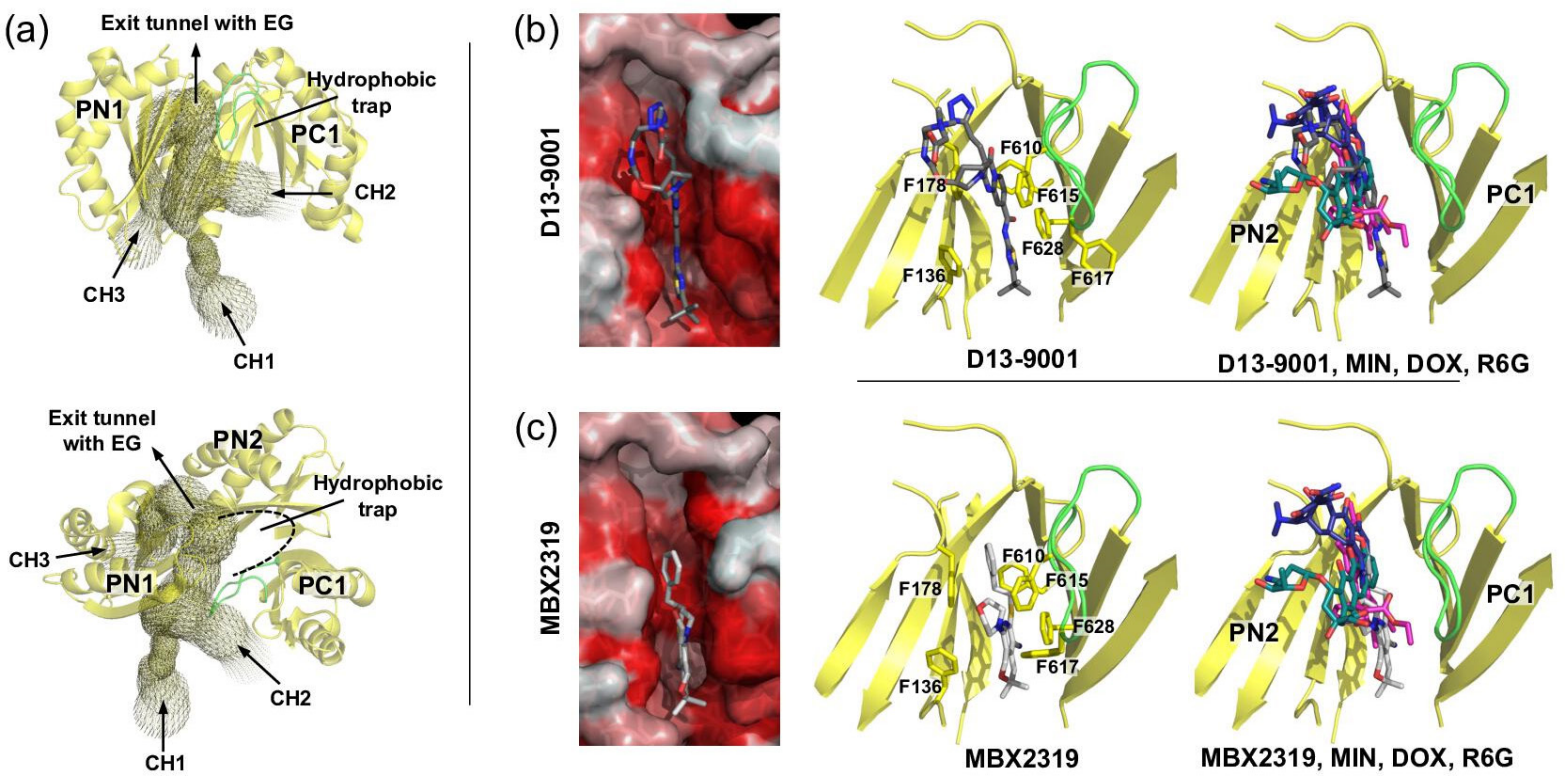
Co-crystal structures illustrating EPI binding to the AcrB DBP and hydrophobic trap. (**a**) Top left: Side view on the periplasmic porter domain (PD) of the AcrB T protomer. Bottom left: Top view on the periplasmic PD of the AcrB T protomer. The PN1, PN2, and PC1 subdomains are shown as yellow cartoon, with the switch loop highlighted in green. The substrate channels are shown in dark mesh, with the three substrate channels CH1, CH2, and CH3 merging in the deep binding pocket (DBP) from where substrates are expelled via the exit tunnel with the exit gate (EG) upon the T to O transition. CH1 and CH2 merge in the access pocket region, whereas CH3 is directly leading to the DBP. The hydrophobic trap (HT) is indicated by a black dotted line. (**b, c**) Crystal co-structures of D13-9001 and MBX2319 bound to the AcrB DBP and HT. Left panel: Side view onto the PN2/PN1 subdomains shown in surface representation and coloured by hydrophobicity (red). Middle and right panel: Side view onto the PN2/PN1 subdomains shown as cartoon (coloured in yellow; β-sheets only). EPIs (middle panel) and superimposed substrates (right panel) are represented as sticks. The carbon atoms of the following side chains, EPIs, and AcrB substrates are coloured as follows: yellow: protein side chains, dark grey: D13-9001 (PDB ID: 3W9H), light grey: MBX2319 (PDB ID: 5ENO), blue: MIN (PDB ID: 4DX5), teal: DOX (PDB ID: 4DX7), pink: R6G (PDB ID: 5ENS), and nitrogen=blue; oxygen=red; sulphur=gold). Binding of both EPIs predominantly involves hydrophobic interactions with residues lining the DBP and HT. Superimposition of EPI and substrate coordinates derived from the AcrBper co-crystal structures illustrates the overlapping binding sites of EPIs and substrates.

Computational and structural studies on PAβN, NMP, D13-9001, and MBX2319 gave valuable insights into the molecular basis of EPI action. Most potent EPIs described so far target the DBP [140, 141, 150, 151]. Co-crystal structures of AcrB/MexB revealed D13-9001 binding to the narrow, hydrophobic pit that branches off the substrate translocation channel within the DBP, i.e. the previously mentioned HT in AcrB [150] (Fig. 12a, b). The hydrophobic tert-butyl thiazolyl aminocarboxyl pyridopyrimidine-moiety of D13-9001 is deeply inserted into the HT, where the pyridopyrimidine and thiazolyl rings make extensive π-π stacking interactions with F178 and F628, respectively. The tetrazole ring and piperidine aceto-amino ethylene ammonio-acetate moiety, however, extend into the substrate translocation channel where they interact with ionic, hydrophilic (N274, R620, Q176 and S180) and aliphatic residues (I277 and L177) [150]. Co-crystal structures of MBX2319 (and later derivates) bound to the AcrB periplasmic domain (AcrBper) display MBX binding to a similar position within the DBP and HT [141] (Fig. 12a, c). The central aromatic pyridine ring of MBX2319 interacts with F628 via π-π stacking. Analogously, the phenyl and morpholinyl groups interact with F178 and F615. F610, and the amphipathic side chains of Y327 and M573, are involved in interactions with the MBX dimethylenesulfide moiety and gem-dimethyl group, respectively. Moreover, the acetamide (MBX3132) and acrylamide (MBX3135) extensions of later MBX analogues were found to be engaged in an extended water-mediated hydrogen bonding network, further contributing to the observed tight binding mode. The binding sites of both D13-9001 and MBX compounds overlap substantially with those reported for the pump substrates MIN, DOX and R6G (Fig. 12b, c). Hence, these EPIs appear to prevent substrate binding to the DBP by steric hindrance [141, 150, 151], as the binding affinities of both D13-9001 and MBX were found to be significantly higher than that of MIN (and most probably other substrates) [140, 141, 151]. Moreover, the tight EPI binding to the HT was suggested to prevent the transition from the T to the O state, thereby hindering the functional rotation of the AcrB trimer [141, 150, 151]. Consistent with this hypothesis, a cryo-EM structure of MBX3132 bound AcrAB-TolC displays the AcrB trimer (predominantly) trapped in the symmetric TTT conformation in which the DBP of each monomer is saturated with and blocked by the EPI [37]. MD simulations suggested a slightly different binding mode for PAβN and NMP within the DBP and HT, in which EPI binding ‘straddles’ the switch loop, most likely resulting in reduced flexibility of the loop motif, which is suggested to hinder substrate translocation into the DBP [140, 144, 151]. Analogously, MBX3132 or D13-9001 are anticipated to interact with switch loop residues F615 and F617 or R620 [141, 150, 151], respectively, suggesting that a similar mechanism might contribute to MBX/D13-9001 activity [166].

All EPIs discussed above target the periplasmic DBP of the RND transporter. However, the proton relay triad within the TMD (residues D407, D408, K940 in AcrB) that is essential for energization of RND-driven efflux constitutes another possible and highly attractive target site. Recently, Plé, Tam *et al.* showed binding of a novel AcrB-specific pyridylpiperazine (PyrPip)-based EPI (BDM88855) (Fig. 6) to a small pocket within the TMD of the AcrB l-protomer (between TMH4, TMH5 and TMH10) [177]. The protonated piperazine moiety of BDM88855 appears to form a critical salt bridge with the D408 side chain, thereby disrupting essential interactions within proton relay network. More recent work [178] describes the optimization of BDM88855 to an inhibitor which was designed to exploit the proximal region at the cytoplasmic rim of the TMD of *K. pneumoniae* AcrB with carboxylic residues D951 as an additional binding partner. This inhibitor, BDM91532, intensifies the interaction in this inhibitor binding pocket and resulted in trapping of the protomer in an intermediate O/L state, as visualized in a single particle cryo-EM structure. This latter BDM inhibitor was shown to reduce bacterial load after 24 h of *K. pneumoniae* infected mice, and holds promise for further *in vivo* use of the BDM series [178]. A similar mechanism of action has previously been suggested for the anti-tubercular drug SQ109 (Fig. 6), which was shown to bind within the TMD proton translocation channel of the HAE-2-type mycolic acid (RND) transporter MmpL3 from *Mycobacterium smegmatis* [179, 180].

Of note, OMF or MFP components of the tripartite complex constitute conceivable targets. Abdali *et al.* [181] identified a set of compounds, among them the dihydroimidazoline NSC60339 and the aminoquinoline NSC33353 (Fig. 6), that were shown to potentiate the activities of NOV and ERY in *E. coli*, with MPC_4_ values in the of range of 12.5–25 µM and 1.56–3.125 µM, respectively [181]. A combination of computational (ensemble docking) and experimental approaches (*in vitro* AcrA binding studies using surface plasmon resonance, as well as *in vivo* limited proteolysis) suggested that both compounds target AcrA, the MFP/PAP component of the tripartite AcrAB-TolC complex. NSC60339 is thought to bind to the hinge region (linking α-hairpin- and lipoyl-domains) of AcrA, thereby constricting conformational flexibility and reducing its ability to transduce signal to AcrB possibly inhibiting functional rotation and efflux [182]. NSC33353, however, is suggested to bind the AcrA membrane proximal (MP) domain (Fig. 2c), presumably interfering with functional interactions between AcrA and AcrB. Both compounds were reported to be AcrB substrates, hence their mode of action could involve interactions with the RND transporter as well [181].

### Inhibitors acting against RND-type efflux pumps from *Acinetobacter baumannii*

Despite the conservation of the binding pocket of BDM88855 in *A. baumannii* RND pumps AdeABC, AdeIJK and AdeFGH, only a few PyrPip derivatives from an extended library (>200 compounds) showed significant antibiotic potentiation effects in *A. baumannii* ATCC17978, and these all were with lower potency compared to the inhibition observed in *E. coli* [183]. For *A. baumannii*, other factors such as PyrPip penetration, multiple pump expression, and slight amino acid side chain variations in the inhibitor binding pocket area may influence the PyrPip activity. By heterologous expression of AdeIJK and AdeFGH pumps in *E. coli*, however, BDM91531, a derivative of BDM88855 (Fig. 6) was found to be the most potent inhibitor with AdeJ as the main target. Clearly, further research is needed to optimize the PyrPip inhibitors for activity in *A. baumannii*.

## Concluding remarks and future research

MDR is attributed to the interplay of multiple mechanisms, one of which is the overexpression of multidrug efflux pumps of the RND superfamily, which recognize and extrude a broad spectrum of structurally diverse compounds. Detailed understanding of the structure-function relationship of these pump systems appears critical for combating MDR. Our knowledge of the substrate promiscuity of RND-type transporters is still poor. The relatively simple and direct comparison between the structures and functional analysis of the AcrB and AdeB transporters shows that the knowledge gained from the presented studies is that molecular determinants for drug recognition of AcrB cannot be simply extrapolated to AdeB and *vice versa*. This is not only on the level of the amino acid side chains involved in the drug binding, but also the conformational states of the protomers within the AcrB and AdeB trimers must be considered.

Directed-evolution experiments in combination with phylogenetic analyses will be useful for the analyses of the molecular determinants of multiple drug binding and transport. Preferably, libraries of a wide range of phylogenetic distant but not too distant HAE-1 family members have to be analysed. Multiple conformational analyses of these selected HAE-1 members, in complex with their tripartite partners, will be feasible using single-particle cryo-EM. In the future, high-resolution *in situ* tripartite structures using cryo-ET and algorithms using an attention network such as AlphaFold will support conformational insight *in vivo*. It will be important to analyse the drug efflux pump properties in their cognate host, since these have evolved in different environments, e.g. AcrAB-TolC in *E. coli*, AdeABC in *A. baumannii*, MexAB-OprM in *P. aeruginosa*. The specific environment of each of the Gram-negative envelopes particularly the OM permeability, must be considered as the entire envelope system modulate efflux activity. Ideally, the combined *in vitro*, *in situ*, and *in vivo* data will have to be fed into molecular dynamics simulation programmes to predict the catalytic efflux cycle in their cognate environment, including multiple efflux pumps. Optimization of EPIs by medicinal chemistry design is highly supported by co-structures of initial inhibitor compounds bound to the efflux pump target site, as shown for AcrB from *E. coli* and *K. pneumoniae*. For EPIs targeting *A. baumannii*, optimization will likewise profit from co-structures, however, permeability across the OM has to be addressed in parallel. Since the role of efflux pumps in the MDR phenotype is often in combination with other resistance mechanisms such as the action of β-lactamases, efflux pumps must be analysed as part of a resistance proteome. The structural and functional data might profit from artificial intelligence algorithms that will reveal correlations between observed efflux activity, efflux pump conformational changes, drug and inhibitor binding, and other resistance mechanisms.

## References

[1] WHO (2017). Global priority list of antibiotic-resistant bacteria to guide research, discovery, and development of new antibiotics.

[2] Tacconelli E, Carrara E, Savoldi A, Harbarth S, Mendelson M (2018). Discovery, research, and development of new antibiotics: the WHO priority list of antibiotic-resistant bacteria and tuberculosis. Lancet Infect Dis.

[3] Murray CJL, Ikuta KS, Sharara F, Swetschinski L, Robles Aguilar G (2022). Global burden of bacterial antimicrobial resistance in 2019: a systematic analysis. The Lancet.

[4] Darby EM, Trampari E, Siasat P, Gaya MS, Alav I (2023). Molecular mechanisms of antibiotic resistance revisited. Nat Rev Microbiol.

[5] Alav I, Sutton JM, Rahman KM (2018). Role of bacterial efflux pumps in biofilm formation. J Antimicrob Chemother.

[6] Wang-Kan X, Blair JMA, Chirullo B, Betts J, La Ragione RM (2017). Lack of AcrB efflux function confers loss of virulence on *Salmonella enterica* serovar typhimurium. mBio.

[7] Nikaido H (2003). Molecular basis of bacterial outer membrane permeability revisited. Microbiol Mol Biol Rev.

[8] Nikaido H, Pagès JM (2012). Broad-specificity efflux pumps and their role in multidrug resistance of Gram-negative bacteria. FEMS Microbiol Rev.

[9] Yoshimura F, Nikaido H (1985). Diffusion of beta-lactam antibiotics through the porin channels of *Escherichia coli* K-12. Antimicrob Agents Chemother.

[10] Vergalli J, Bodrenko IV, Masi M, Moynié L, Acosta-Gutiérrez S (2020). Porins and small-molecule translocation across the outer membrane of Gram-negative bacteria. Nat Rev Microbiol.

[11] Pagès JM, James CE, Winterhalter M (2008). The porin and the permeating antibiotic: a selective diffusion barrier in Gram-negative bacteria. Nat Rev Microbiol.

[12] Sugawara E, Nikaido H (2012). OmpA is the principal nonspecific slow porin of *Acinetobacter baumannii*. J Bacteriol.

[13] Nikaido H (1996). Multidrug efflux pumps of gram-negative bacteria. J Bacteriol.

[14] Shuster Y, Steiner-Mordoch S, Alon Cudkowicz N, Schuldiner S (2016). A transporter interactome is essential for the acquisition of antimicrobial resistance to antibiotics. PLoS One.

[15] Rahman T, Yarnall B, Doyle DA (2017). Efflux drug transporters at the forefront of antimicrobial resistance. Eur Biophys J.

[16] Tal N, Schuldiner S (2009). A coordinated network of transporters with overlapping specificities provides a robust survival strategy. Proc Natl Acad Sci U S A.

[17] Sulavik MC, Houseweart C, Cramer C, Jiwani N, Murgolo N (2001). Antibiotic susceptibility profiles of *Escherichia coli* strains lacking multidrug efflux pump genes. Antimicrob Agents Chemother.

[18] Nishino K, Yamaguchi A (2001). Analysis of a complete library of putative drug transporter genes in *Escherichia coli*. J Bacteriol.

[19] de Cristóbal RE, Vincent PA, Salomón RA (2006). Multidrug resistance pump AcrAB-TolC is required for high-level, Tet(A)-mediated tetracycline resistance in *Escherichia coli*. J Antimicrob Chemother.

[20] Teelucksingh T, Thompson LK, Zhu S, Kuehfuss NM, Goetz JA (2022). A genetic platform to investigate the functions of bacterial drug efflux pumps. Nat Chem Biol.

[21] Marchand I, Damier-Piolle L, Courvalin P, Lambert T (2004). Expression of the RND-type efflux pump AdeABC in *Acinetobacter baumannii* is regulated by the AdeRS two-component system. Antimicrob Agents Chemother.

[22] Coyne S, Rosenfeld N, Lambert T, Courvalin P, Périchon B (2010). Overexpression of resistance-nodulation-cell division pump AdeFGH confers multidrug resistance in *Acinetobacter baumannii*. Antimicrob Agents Chemother.

[23] Cohen SP, Hächler H, Levy SB (1993). Genetic and functional analysis of the multiple antibiotic resistance (mar) locus in *Escherichia coli*. J Bacteriol.

[24] Webber MA, Talukder A, Piddock LJV (2005). Contribution of mutation at amino acid 45 of AcrR to acrB expression and ciprofloxacin resistance in clinical and veterinary *Escherichia coli* isolates. Antimicrob Agents Chemother.

[25] Weston N, Sharma P, Ricci V, Piddock LJV (2018). Regulation of the AcrAB-TolC efflux pump in Enterobacteriaceae. Res Microbiol.

[26] Du D, Wang-Kan X, Neuberger A, van Veen HW, Pos KM (2018). Multidrug efflux pumps: structure, function and regulation. Nat Rev Microbiol.

[27] Blair JMA, Bavro VN, Ricci V, Modi N, Cacciotto P (2015). AcrB drug-binding pocket substitution confers clinically relevant resistance and altered substrate specificity. Proc Natl Acad Sci U S A.

[28] Elkins C, Nikaido H (2002). Substrate Speci city of the RND-type multidrug EF ux pumps AcrB and AcrD of. J Bacteriol.

[29] Tikhonova EB, Wang Q, Zgurskaya HI (2002). Chimeric analysis of the multicomponent multidrug efflux transporters from gram-negative bacteria. J Bacteriol.

[30] Eda S, Maseda H, Nakae T (2003). An elegant means of self-protection in gram-negative bacteria by recognizing and extruding xenobiotics from the periplasmic space. J Biol Chem.

[31] Nikaido H (2009). Multidrug resistance in bacteria. Annu Rev Biochem.

[32] Guan L, Nakae T (2001). Identification of essential charged residues in transmembrane segments of the multidrug transporter MexB of *Pseudomonas aeruginosa*. J Bacteriol.

[33] Seeger MA, von Ballmoos C, Verrey F, Pos KM (2009). Crucial role of Asp408 in the proton translocation pathway of multidrug transporter AcrB: evidence from site-directed mutagenesis and carbodiimide labeling. Biochemistry.

[34] Su C-C, Li M, Gu R, Takatsuka Y, McDermott G (2006). Conformation of the AcrB multidrug efflux pump in mutants of the putative proton relay pathway. J Bacteriol.

[35] Alav I, Bavro VN, Blair JMA (2022). A role for the periplasmic adaptor protein AcrA in vetting substrate access to the RND efflux transporter AcrB. Sci Rep.

[36] Daury L, Orange F, Taveau J-C, Verchère A, Monlezun L (2016). Tripartite assembly of RND multidrug efflux pumps. Nat Commun.

[37] Wang Z, Fan G, Hryc CF, Blaza JN, Serysheva II (2017). An allosteric transport mechanism for the AcrAB-TolC multidrug efflux pump. Elife.

[38] Tsutsumi K, Yonehara R, Ishizaka-Ikeda E, Miyazaki N, Maeda S (2019). Structures of the wild-type MexAB-OprM tripartite pump reveal its complex formation and drug efflux mechanism. Nat Commun.

[39] Glavier M, Puvanendran D, Salvador D, Decossas M, Phan G (2020). Antibiotic export by MexB multidrug efflux transporter is allosterically controlled by a MexA-OprM chaperone-like complex. Nat Commun.

[40] Du D, Wang Z, James NR, Voss JE, Klimont E (2014). Structure of the AcrAB-TolC multidrug efflux pump. Nature.

[41] Hobbs EC, Yin X, Paul BJ, Astarita JL, Storz G (2012). Conserved small protein associates with the multidrug efflux pump AcrB and differentially affects antibiotic resistance. Proc Natl Acad Sci U S A.

[42] Alav I, Kobylka J, Kuth MS, Pos KM, Picard M (2021). Structure, assembly, and function of tripartite efflux and type 1 secretion systems in Gram-negative bacteria. Chem Rev.

[43] Zgurskaya HI, Walker JK, Parks JM, Rybenkov VV (2021). Multidrug efflux pumps and the two-faced janus of substrates and inhibitors. Acc Chem Res.

[44] Krishnamoorthy G, Leus IV, Weeks JW, Wolloscheck D, Rybenkov VV (2017). Synergy between active efflux and outer membrane diffusion defines rules of antibiotic permeation into Gram-negative bacteria. mBio.

[45] Saier MH, Tran CV, Barabote RD (2006). TCDB: the transporter classification database for membrane transport protein analyses and information. Nucleic Acids Res.

[46] Saier MH, Reddy VS, Tsu BV, Ahmed MS, Li C (2016). The Transporter Classification Database (TCDB): recent advances. Nucleic Acids Res.

[47] Saier MH, Reddy VS, Moreno-Hagelsieb G, Hendargo KJ, Zhang Y (2021). The Transporter Classification Database (TCDB): 2021 update. Nucleic Acids Res.

[48] Thomas C, Tampé R (2020). Structural and mechanistic principles of ABC transporters. Annu Rev Biochem.

[49] Kornelsen V, Kumar A (2021). Update on multidrug resistance efflux pumps in Acinetobacter spp. Antimicrob Agents Chemother.

[50] Kim J, Cater RJ, Choy BC, Mancia F (2021). Structural Insights into transporter-mediated drug resistance in infectious diseases. J Mol Biol.

[51] Henderson PJF, Maher C, Elbourne LDH, Eijkelkamp BA, Paulsen IT (2021). Physiological functions of bacterial “Multidrug” efflux pumps. Chem Rev.

[52] Tseng TT, Gratwick KS, Kollman J, Park D, Nies DH (1999). The RND permease superfamily: an ancient, ubiquitous and diverse family that includes human disease and development proteins. J Mol Microbiol Biotechnol.

[53] Nikaido H (2018). RND transporters in the living world. Res Microbiol.

[54] Tsukazaki T (2018). Structure-based working model of SecDF, a proton-driven bacterial protein translocation factor. FEMS Microbiol Lett.

[55] Pogliano JA, Beckwith J (1994). SecD and SecF facilitate protein export in *Escherichia coli*. EMBO J.

[56] Varela C, Rittmann D, Singh A, Krumbach K, Bhatt K (2012). MmpL genes are associated with mycolic acid metabolism in mycobacteria and corynebacteria. Chem Biol.

[57] Adams O, Deme JC, Parker JL, Fowler PW, Lea SM (2021). Cryo-EM structure and resistance landscape of M. tuberculosis MmpL3: an emergent therapeutic target. Structure.

[58] Kumar N, Su C-C, Chou T-H, Radhakrishnan A, Delmar JA (2017). Crystal structures of the *Burkholderia multivorans* hopanoid transporter HpnN. Proc Natl Acad Sci U S A.

[59] Maher C, Hassan KA (2023). The Gram-negative permeability barrier: tipping the balance of the in and the out. mBio.

[60] Kobylka J, Kuth MS, Müller RT, Geertsma ER, Pos KM (2020). AcrB: a mean, keen, drug efflux machine. Ann N Y Acad Sci.

[61] Klenotic PA, Moseng MA, Morgan CE, Yu EW (2021). Structural and functional diversity of resistance-nodulation-cell division transporters. Chem Rev.

[62] Murakami S, Nakashima R, Yamashita E, Yamaguchi A (2002). Crystal structure of bacterial multidrug efflux transporter AcrB. Nature.

[63] Eicher T, Seeger MA, Anselmi C, Zhou W, Brandstätter L (2014). Coupling of remote alternating-access transport mechanisms for protons and substrates in the multidrug efflux pump AcrB. Elife.

[64] Takatsuka Y, Nikaido H (2006). Threonine-978 in the transmembrane segment of the multidrug efflux pump AcrB of *Escherichia coli* is crucial for drug transport as a probable component of the proton relay network. J Bacteriol.

[65] Kim J-S, Jeong H, Song S, Kim H-Y, Lee K (2015). Structure of the tripartite multidrug efflux pump AcrAB-TolC suggests an alternative assembly mode. Mol Cells.

[66] Jeong H, Kim J-S, Song S, Shigematsu H, Yokoyama T (2016). Pseudoatomic structure of the tripartite multidrug efflux pump AcrAB-TolC reveals the intermeshing cogwheel-like interaction between AcrA and TolC. Structure.

[67] Tsutsumi K, Yonehara R, Ishizaka-Ikeda E, Miyazaki N, Maeda S (2019). Structures of the wild-type MexAB-OprM tripartite pump reveal its complex formation and drug efflux mechanism. Nat Commun.

[68] Glavier M, Puvanendran D, Salvador D, Decossas M, Phan G (2020). Antibiotic export by MexB multidrug efflux transporter is allosterically controlled by a MexA-OprM chaperone-like complex. Nat Commun.

[69] Takatsuka Y, Nikaido H (2007). Site-directed disulfide cross-linking shows that cleft flexibility in the periplasmic domain is needed for the multidrug efflux pump AcrB of *Escherichia coli*. J Bacteriol.

[70] Rahman MM, Matsuo T, Ogawa W, Koterasawa M, Kuroda T (2007). Molecular cloning and characterization of all RND-type efflux transporters in Vibrio cholerae non-O1. Microbiol Immunol.

[71] Kim HS, Nagore D, Nikaido H (2010). Multidrug efflux pump MdtBC of *Escherichia coli* is active only as a B2C heterotrimer. J Bacteriol.

[72] Kim H-S, Nikaido H (2012). Different functions of MdtB and MdtC subunits in the heterotrimeric efflux transporter MdtB(2)C complex of *Escherichia coli*. Biochemistry.

[73] Sennhauser G, Bukowska MA, Briand C, Grütter MG (2009). Crystal structure of the multidrug exporter MexB from *Pseudomonas aeruginosa*. J Mol Biol.

[74] Bolla JR, Su C-C, Do SV, Radhakrishnan A, Kumar N (2014). Crystal structure of the Neisseria gonorrhoeae MtrD inner membrane multidrug efflux pump. PLoS ONE.

[75] Su C-C, Yin L, Kumar N, Dai L, Radhakrishnan A (2017). Structures and transport dynamics of a *Campylobacter jejuni* multidrug efflux pump. Nat Commun.

[76] Su C-C, Morgan CE, Kambakam S, Rajavel M, Scott H (2019). Cryo-electron microscopy structure of an *Acinetobacter baumannii* multidrug efflux pump. mBio.

[77] Morgan CE, Glaza P, Leus IV, Trinh A, Su C-C (2021). Cryoelectron microscopy structures of AdeB illuminate mechanisms of simultaneous binding and exporting of substrates. mBio.

[78] Ornik-Cha A, Wilhelm J, Kobylka J, Sjuts H, Vargiu AV (2021). Structural and functional analysis of the promiscuous AcrB and AdeB efflux pumps suggests different drug binding mechanisms. Nat Commun.

[79] Johnson RM, Fais C, Parmar M, Cheruvara H, Marshall RL (2020). Cryo-EM structure and molecular dynamics analysis of the fluoroquinolone resistant mutant of the AcrB transporter from *Salmonella*. Microorganisms.

[80] Fabre L, Ntreh AT, Yazidi A, Leus IV, Weeks JW (2021). A “Drug Sweeping” state of the TriABC triclosan efflux pump from *Pseudomonas aeruginosa*. Structure.

[81] Lyu M, Moseng MA, Reimche JL, Holley CL, Dhulipala V (2020). Cryo-EM structures of a gonococcal multidrug efflux pump illuminate a mechanism of drug recognition and resistance. mBio.

[82] Zhang Z, Morgan CE, Bonomo RA, Yu EW (2021). Cryo-EM determination of Eravacycline-bound structures of the Baumannii. mBio.

[83] Zhang Z, Morgan CE, Cui M, Yu EW, Engelman AN (2023). Cryo-EM structures of AcrD illuminate a mechanism for capturing Aminoglycosides from its central cavity. mBio.

[84] Bharatham N, Bhowmik P, Aoki M, Okada U, Sharma S (2021). Structure and function relationship of OqxB efflux pump from *Klebsiella pneumoniae*. Nat Commun.

[85] Kato T, Okada U, Hung L-W, Yamashita E, Kim H-B (2023). Crystal structures of multidrug efflux transporters from *Burkholderia pseudomallei* suggest details of transport mechanism. Proc Natl Acad Sci U S A.

[86] Deininger KNW, Horikawa A, Kitko RD, Tatsumi R, Rosner JL (2011). A requirement of TolC and MDR efflux pumps for acid adaptation and GadAB induction in *Escherichia coli*. PLoS One.

[87] Schaffner SH, Lee AV, Pham MTN, Kassaye BB, Li H (2021). Extreme acid modulates fitness trade-offs of multidrug efflux pumps MdtEF-TolC and AcrAB-TolC in *Escherichia coli* K-12. Appl Environ Microbiol.

[88] Zhang Y, Xiao M, Horiyama T, Zhang Y, Li X (2011). The multidrug efflux pump MdtEF protects against nitrosative damage during the anaerobic respiration in *Escherichia coli*. J Biol Chem.

[89] Rosenberg EY, Ma D, Nikaido H (2000). AcrD of *Escherichia coli* is an aminoglycoside efflux pump. J Bacteriol.

[90] Ramaswamy VK, Vargiu AV, Malloci G, Dreier J, Ruggerone P (2017). Molecular rationale behind the differential substrate specificity of bacterial RND multi-drug transporters. Sci Rep.

[91] Cuesta Bernal J, El-Delik J, Göttig S, Pos KM (2021). Characterization and molecular determinants for β-lactam specificity of the multidrug efflux pump AcrD from *Salmonella typhimurium*. Antibiotics (Basel).

[92] Nikaido H, Basina M, Nguyen V, Rosenberg EY (1998). Multidrug efflux pump AcrAB of Salmonella typhimurium excretes only those beta-lactam antibiotics containing lipophilic side chains. J Bacteriol.

[93] Zgurskaya HI, López CA, Gnanakaran S (2015). Permeability barrier of Gram-negative cell envelopes and approaches to bypass it. ACS Infect Dis.

[94] Lee A, Mao W, Warren MS, Mistry A, Hoshino K (2000). Interplay between efflux pumps may provide either additive or multiplicative effects on drug resistance. J Bacteriol.

[95] Foong WE, Wilhelm J, Tam H-K, Pos KM (2020). Tigecycline efflux in Acinetobacter baumannii is mediated by TetA in synergy with RND-type efflux transporters. J Antimicrob Chemother.

[96] Pos KM (2009). Drug transport mechanism of the AcrB efflux pump. Biochim Biophys Acta.

[97] Brandstätter L, Sokolova L, Eicher T, Seeger MA, Briand C (2011). Analysis of AcrB and AcrB/DARPin ligand complexes by LILBID MS. Biochim Biophys Acta.

[98] Yu L, Lu W, Wei Y, Sandler SJ (2011). AcrB trimer stability and efflux activity, insight from mutagenesis studies. PLoS ONE.

[99] Seeger MA, von Ballmoos C, Eicher T, Brandstätter L, Verrey F (2008). Engineered disulfide bonds support the functional rotation mechanism of multidrug efflux pump AcrB. Nat Struct Mol Biol.

[100] Murakami S, Tamura N, Saito A, Hirata T, Yamaguchi A (2004). Extramembrane central pore of multidrug exporter AcrB in *Escherichia coli* plays an important role in drug transport. J Biol Chem.

[101] Qiu W, Fu Z, Xu GG, Grassucci RA, Zhang Y (2018). Structure and activity of lipid bilayer within a membrane-protein transporter. Proc Natl Acad Sci U S A.

[102] Murakami S, Nakashima R, Yamashita E, Matsumoto T, Yamaguchi A (2006). Crystal structures of a multidrug transporter reveal a functionally rotating mechanism. Nature.

[103] Seeger MA, Schiefner A, Eicher T, Verrey F, Diederichs K (2006). Structural asymmetry of AcrB trimer suggests a peristaltic pump mechanism. Science.

[104] Sennhauser G, Amstutz P, Briand C, Storchenegger O, Grütter MG (2007). Drug export pathway of multidrug exporter AcrB revealed by DARPin inhibitors. PLoS Biol.

[105] Nakashima R, Sakurai K, Yamasaki S, Nishino K, Yamaguchi A (2011). Structures of the multidrug exporter AcrB reveal a proximal multisite drug-binding pocket. Nature.

[106] Eicher T, Cha H, Seeger MA, Brandstätter L, El-Delik J (2012). Transport of drugs by the multidrug transporter AcrB involves an access and a deep binding pocket that are separated by a switch-loop. Proc Natl Acad Sci U S A.

[107] Boyer PD (1997). The ATP synthase--a splendid molecular machine. Annu Rev Biochem.

[108] Takatsuka Y, Nikaido H (2009). Covalently linked trimer of the AcrB multidrug efflux pump provides support for the functional rotating mechanism. J Bacteriol.

[109] Nagano K, Nikaido H (2009). Kinetic behavior of the major multidrug efflux pump AcrB of *Escherichia coli*. Proc Natl Acad Sci U S A.

[110] Lim SP, Nikaido H (2010). Kinetic parameters of efflux of penicillins by the multidrug efflux transporter AcrAB-TolC of *Escherichia coli*. Antimicrob Agents Chemother.

[111] Müller RT, Pos KM (2015). The assembly and disassembly of the AcrAB-TolC three-component multidrug efflux pump. Biol Chem.

[112] Yao X-Q, Kenzaki H, Murakami S, Takada S (2010). Drug export and allosteric coupling in a multidrug transporter revealed by molecular simulations. Nat Commun.

[113] Zwama M, Yamasaki S, Nakashima R, Sakurai K, Nishino K (2018). Multiple entry pathways within the efflux transporter AcrB contribute to multidrug recognition. Nat Commun.

[114] Oswald C, Tam HK, Pos KM (2016). Transport of lipophilic carboxylates is mediated by transmembrane helix 2 in multidrug transporter AcrB. Nat Commun.

[115] Tam H-K, Malviya VN, Foong W-E, Herrmann A, Malloci G (2020). Binding and transport of carboxylated drugs by the multidrug transporter AcrB. J Mol Biol.

[116] Tam H-K, Foong WE, Oswald C, Herrmann A, Zeng H (2021). Allosteric drug transport mechanism of multidrug transporter AcrB. Nat Commun.

[117] Zgurskaya HI, Nikaido H (1999). Bypassing the periplasm: reconstitution of the AcrAB multidrug efflux pump of *Escherichia coli*. Proc Natl Acad Sci U S A.

[118] Zwama M, Hayashi K, Sakurai K, Nakashima R, Kitagawa K (2017). Hoisting-loop in bacterial multidrug exporter AcrB is a highly flexible hinge that enables the large motion of the subdomains. Front Microbiol.

[119] Yamane T, Murakami S, Ikeguchi M (2013). Functional rotation induced by alternating protonation states in the multidrug transporter AcrB: all-atom molecular dynamics simulations. Biochemistry.

[120] Yue Z, Chen W, Zgurskaya HI, Shen J (2017). Constant pH molecular dynamics reveals how proton release drives the conformational transition of a transmembrane efflux pump. J Chem Theory Comput.

[121] Seeger MA, Diederichs K, Eicher T, Brandstätter L, Schiefner A (2008). The AcrB efflux pump: conformational cycling and peristalsis lead to multidrug resistance. Curr Drug Targets.

[122] Yamaguchi A, Nakashima R, Sakurai K (2015). Structural basis of RND-type multidrug exporters. Front Microbiol.

[123] Nishino K, Yamada J, Hirakawa H, Hirata T, Yamaguchi A (2003). Roles of TolC-dependent multidrug transporters of *Escherichia coli* in resistance to beta-lactams. Antimicrob Agents Chemother.

[124] Aires JR, Köhler T, Nikaido H, Plésiat P (1999). Involvement of an active efflux system in the natural resistance of *Pseudomonas aeruginosa* to aminoglycosides. Antimicrob Agents Chemother.

[125] Masuda N, Sakagawa E, Ohya S, Gotoh N, Tsujimoto H (2000). Substrate specificities of MexAB-OprM, MexCD-OprJ, and MexXY-oprM efflux pumps in *Pseudomonas aeruginosa*. Antimicrob Agents Chemother.

[126] Magnet S, Courvalin P, Lambert T (2001). Resistance-nodulation-cell division-type efflux pump involved in aminoglycoside resistance in *Acinetobacter baumannii* strain BM4454. Antimicrob Agents Chemother.

[127] Rajamohan G, Srinivasan VB, Gebreyes WA (2010). Novel role of Acinetobacter baumannii RND efflux transporters in mediating decreased susceptibility to biocides. J Antimicrob Chemother.

[128] Coyne S, Courvalin P, Périchon B (2011). Efflux-mediated antibiotic resistance in Acinetobacter spp. Antimicrob Agents Chemother.

[129] Sugawara E, Nikaido H (2014). Properties of AdeABC and AdeIJK efflux systems of *Acinetobacter baumannii* compared with those of the AcrAB-TolC system of *Escherichia coli*. Antimicrob Agents Chemother.

[130] Yoon E-J, Chabane YN, Goussard S, Snesrud E, Courvalin P (2015). Contribution of resistance-nodulation-cell division efflux systems to antibiotic resistance and biofilm formation in *Acinetobacter baumannii*. mBio.

[131] Richmond GE, Evans LP, Anderson MJ, Wand ME, Bonney LC (2016). The *Acinetobacter baumannii* two-component system AdeRS regulates genes required for multidrug efflux, biofilm formation, and virulence in a strain-specific manner. mBio.

[132] Yu EW, McDermott G, Zgurskaya HI, Nikaido H, Koshland DE (2003). Structural basis of multiple drug-binding capacity of the AcrB multidrug efflux pump. Science.

[133] Yu EW, Aires JR, McDermott G, Nikaido H (2005). A periplasmic drug-binding site of the AcrB multidrug efflux pump: a crystallographic and site-directed mutagenesis study. J Bacteriol.

[134] Törnroth-Horsefield S, Gourdon P, Horsefield R, Brive L, Yamamoto N (2007). Crystal structure of AcrB in complex with a single transmembrane subunit reveals another twist. Structure.

[135] Drew D, Klepsch MM, Newstead S, Flaig R, De Gier J-W (2008). The structure of the efflux pump AcrB in complex with bile acid. Mol Membr Biol.

[136] Neyfakh AA (2002). Mystery of multidrug transporters: the answer can be simple. Mol Microbiol.

[137] Zwama M, Yamaguchi A (2018). Molecular mechanisms of AcrB-mediated multidrug export. Res Microbiol.

[138] Vargiu AV, Ramaswamy VK, Malloci G, Malvacio I, Atzori A (2018). Computer simulations of the activity of RND efflux pumps. Res Microbiol.

[139] Takatsuka Y, Chen C, Nikaido H (2010). Mechanism of recognition of compounds of diverse structures by the multidrug efflux pump AcrB of Escherichia coli. Proc Natl Acad Sci U S A.

[140] Vargiu AV, Nikaido H (2012). Multidrug binding properties of the AcrB efflux pump characterized by molecular dynamics simulations. Proc Natl Acad Sci U S A.

[141] Sjuts H, Vargiu AV, Kwasny SM, Nguyen ST, Kim H-S (2016). Molecular basis for inhibition of AcrB multidrug efflux pump by novel and powerful pyranopyridine derivatives. Proc Natl Acad Sci U S A.

[142] Pos KM (2024). RND multidrug efflux transporters: similar appearances, diverse actions. J Bacteriol.

[143] Ababou A, Koronakis V (2016). Structures of gate loop variants of the AcrB drug efflux pump bound by erythromycin substrate. PLoS ONE.

[144] Cha H, Müller RT, Pos KM (2014). Switch-loop flexibility affects transport of large drugs by the promiscuous AcrB multidrug efflux transporter. Antimicrob Agents Chemother.

[145] Müller RT, Travers T, Cha H-J, Phillips JL, Gnanakaran S (2017). Switch loop flexibility affects substrate transport of the AcrB efflux pump. J Mol Biol.

[146] Ababou A (2018). New insights into the structural and functional involvement of the gate loop in AcrB export activity. Biochim Biophys Acta Proteins Proteom.

[147] Long F, Su C-C, Zimmermann MT, Boyken SE, Rajashankar KR (2010). Crystal structures of the CusA efflux pump suggest methionine-mediated metal transport. Nature.

[148] Su C-C, Long F, Lei H-T, Bolla JR, Do SV (2012). Charged amino acids (R83, E567, D617, E625, R669, and K678) of CusA are required for metal ion transport in the Cus efflux system. J Mol Biol.

[149] Leus IV, Roberts SR, Trinh A, W Yu E, Zgurskaya HI (2024). Nonadditive functional interactions between ligand-binding sites of the multidrug efflux pump AdeB from *Acinetobacter baumannii*. J Bacteriol.

[150] Nakashima R, Sakurai K, Yamasaki S, Hayashi K, Nagata C (2013). Structural basis for the inhibition of bacterial multidrug exporters. Nature.

[151] Vargiu AV, Ruggerone P, Opperman TJ, Nguyen ST, Nikaido H (2014). Molecular mechanism of MBX2319 inhibition of *Escherichia coli* AcrB multidrug efflux pump and comparison with other inhibitors. Antimicrob Agents Chemother.

[152] Bohnert JA, Schuster S, Seeger MA, Fähnrich E, Pos KM (2008). Site-directed mutagenesis reveals putative substrate binding residues in the *Escherichia coli* RND efflux pump AcrB. J Bacteriol.

[153] Vargiu AV, Collu F, Schulz R, Pos KM, Zacharias M (2011). Effect of the F610A mutation on substrate extrusion in the AcrB transporter: explanation and rationale by molecular dynamics simulations. J Am Chem Soc.

[154] Yao XQ, Kimura N, Murakami S, Takada S (2013). Drug uptake pathways of multidrug transporter AcrB studied by molecular simulations and site-directed mutagenesis experiments. J Am Chem Soc.

[155] Verchère A, Dezi M, Adrien V, Broutin I, Picard M (2015). In vitro transport activity of the fully assembled MexAB-OprM efflux pump from *Pseudomonas aeruginosa*. Nat Commun.

[156] Picard M, Tikhonova EB, Broutin I, Lu S, Verchère A, Yamaguchi A, Nishino K Bacterial Multidrug Exporters.

[157] Kinana AD, Vargiu AV, May T, Nikaido H (2016). Aminoacyl β-naphthylamides as substrates and modulators of AcrB multidrug efflux pump. Proc Natl Acad Sci U S A.

[158] Masi M, Réfregiers M, Pos KM, Pagès J-M (2017). Mechanisms of envelope permeability and antibiotic influx and efflux in Gram-negative bacteria. Nat Microbiol.

[159] Masi M, Dumont E, Vergalli J, Pajovic J, Réfrégiers M (2018). Fluorescence enlightens RND pump activity and the intrabacterial concentration of antibiotics. Res Microbiol.

[160] Schuster S, Vavra M, Wirth DAN, Kern WV (2024). Comparative reassessment of AcrB efflux inhibitors reveals differential impact of specific pump mutations on the activity of potent compounds. Microbiol Spectr.

[161] Piddock LJV (2006). Clinically relevant chromosomally encoded multidrug resistance efflux pumps in bacteria. Clin Microbiol Rev.

[162] Opperman TJ, Nguyen ST (2015). Recent advances toward a molecular mechanism of efflux pump inhibition. Front Microbiol.

[163] Lomovskaya O, Warren MS, Lee A, Galazzo J, Fronko R (2001). Identification and characterization of inhibitors of multidrug resistance efflux pumps in *Pseudomonas aeruginosa*: novel agents for combination therapy. Antimicrob Agents Chemother.

[164] Kvist M, Hancock V, Klemm P (2008). Inactivation of efflux pumps abolishes bacterial biofilm formation. Appl Environ Microbiol.

[165] Nishino K, Latifi T, Groisman EA (2006). Virulence and drug resistance roles of multidrug efflux systems of Salmonella enterica serovar Typhimurium. Mol Microbiol.

[166] Aron Z, Opperman TJ (2018). The hydrophobic trap-the Achilles heel of RND efflux pumps. Res Microbiol.

[167] Compagne N, Vieira Da Cruz A, Müller RT, Hartkoorn RC, Flipo M (2023). Update on the discovery of efflux pump Inhibitors against critical priority Gram-negative bacteria. Antibiotics (Basel).

[168] Renau TE, Léger R, Flamme EM, Sangalang J, She MW (1999). Inhibitors of efflux pumps in *Pseudomonas aeruginosa* potentiate the activity of the fluoroquinolone antibacterial levofloxacin. J Med Chem.

[169] Watkins WJ, Landaverry Y, Léger R, Litman R, Renau TE (2003). The relationship between physicochemical properties, in vitro activity and pharmacokinetic profiles of analogues of diamine-containing efflux pump inhibitors. Bioorg Med Chem Lett.

[170] Lomovskaya O, Bostian KA (2006). Practical applications and feasibility of efflux pump inhibitors in the clinic--A vision for applied use. Biochem Pharmacol.

[171] Schumacher A, Steinke P, Bohnert JA, Akova M, Jonas D (2006). Effect of 1-(1-naphthylmethyl)-piperazine, a novel putative efflux pump inhibitor, on antimicrobial drug susceptibility in clinical isolates of Enterobacteriaceae other than *Escherichia coli*. J Antimicrob Chemother.

[172] Bohnert JA, Kern WV (2005). Selected arylpiperazines are capable of reversing multidrug resistance in Escherichia coli overexpressing RND efflux pumps. Antimicrob Agents Chemother.

[173] Nakayama K, Ishida Y, Ohtsuka M, Kawato H, Yoshida K ichi (2003). MexAB-OprM-specific efflux pump inhibitors in *Pseudomonas aeruginosa*. Part 1: discovery and early strategies for lead optimization. Bioorg Med Chem Lett.

[174] Yoshida K-I, Nakayama K, Ohtsuka M, Kuru N, Yokomizo Y (2007). MexAB-OprM specific efflux pump inhibitors in *Pseudomonas aeruginosa*. Part 7: highly soluble and in vivo active quaternary ammonium analogue D13-9001, a potential preclinical candidate. Bioorg Med Chem.

[175] Opperman TJ, Kwasny SM, Kim H-S, Nguyen ST, Houseweart C (2014). Characterization of a novel pyranopyridine inhibitor of the AcrAB efflux pump of *Escherichia coli*. Antimicrob Agents Chemother.

[176] Nguyen ST, Kwasny SM, Ding X, Cardinale SC, McCarthy CT (2015). Structure-activity relationships of a novel pyranopyridine series of Gram-negative bacterial efflux pump inhibitors. Bioorg Med Chem.

[177] Plé C, Tam H-K, Vieira Da Cruz A, Compagne N, Jiménez-Castellanos J-C (2022). Pyridylpiperazine-based allosteric inhibitors of RND-type multidrug efflux pumps. Nat Commun.

[178] Vieira Da Cruz A, Jiménez-Castellanos J-C, Börnsen C, Van Maele L, Compagne N (2024). Pyridylpiperazine efflux pump inhibitor boosts in vivo antibiotic efficacy against K. pneumoniae. EMBO Mol Med.

[179] Zhang B, Li J, Yang X, Wu L, Zhang J (2019). Crystal structures of membrane transporter MmpL3, an Anti-TB drug target. Cell.

[180] Li W, Stevens CM, Pandya AN, Darzynkiewicz Z, Bhattarai P (2019). Direct inhibition of MmpL3 by novel antitubercular compounds. ACS Infect Dis.

[181] Abdali N, Parks JM, Haynes KM, Chaney JL, Green AT (2017). Reviving antibiotics: efflux pump inhibitors that interact with AcrA, a membrane fusion protein of the AcrAB-TolC multidrug efflux pump. ACS Infect Dis.

[182] Russell Lewis B, Uddin MR, Moniruzzaman M, Kuo KM, Higgins AJ (2023). Conformational restriction shapes the inhibition of a multidrug efflux adaptor protein. Nat Commun.

[183] Jiménez-Castellanos J-C, Pradel E, Compagne N, Vieira Da Cruz A, Flipo M (2023). Characterization of pyridylpiperazine-based efflux pump inhibitors for *Acinetobacter baumannii*. JAC Antimicrob Resist.

